# Brain Transcriptional and Epigenetic Associations with Autism

**DOI:** 10.1371/journal.pone.0044736

**Published:** 2012-09-12

**Authors:** Matthew R. Ginsberg, Robert A. Rubin, Tatiana Falcone, Angela H. Ting, Marvin R. Natowicz

**Affiliations:** 1 Cleveland Clinic Lerner College of Medicine, Cleveland, Ohio, United States of America; 2 Department of Mathematics, Whittier College, Whittier, California, United States of America; 3 Neurological Institute, Cleveland Clinic, Cleveland, Ohio, United States of America; 4 Genomic Medicine Institute, Cleveland Clinic, Cleveland, Ohio, United States of America; 5 Pathology and Laboratory Medicine and Pediatrics Institutes, Cleveland Clinic, Cleveland, Ohio, United States of America; University of Insubria, Italy

## Abstract

**Background:**

Autism is a common neurodevelopmental syndrome. Numerous rare genetic etiologies are reported; most cases are idiopathic.

**Methodology/Principal Findings:**

To uncover important gene dysregulation in autism we analyzed carefully selected idiopathic autistic and control cerebellar and BA19 (occipital) brain tissues using high resolution whole genome gene expression and whole genome DNA methylation microarrays. No changes in DNA methylation were identified in autistic brain but gene expression abnormalities in two areas of metabolism were apparent: down-regulation of genes of mitochondrial oxidative phosphorylation and of protein translation. We also found associations between specific behavioral domains of autism and specific brain gene expression modules related to myelin/myelination, inflammation/immune response and purinergic signaling.

**Conclusions/Significance:**

This work highlights two largely unrecognized molecular pathophysiological themes in autism and suggests differing molecular bases for autism behavioral endophenotypes.

## Introduction

Autism comprises a spectrum of neurodevelopmental disorders characterized by social impairments, language or communication difficulties, and restricted, stereotyped and repetitive behaviors. It is highly prevalent, has an approximately 4∶1 male:female predominance, and there is highly variable phenotypic involvement in each of the core symptom domains in both genders [1]. Autism has high heritability, with numerous uncommon or rare genetic etiologies noted in about 10% of affected individuals; most cases are idiopathic [2]–[4]. Recent whole-exome sequencing studies have had limited success in identifying strong effect mutations in many persons with autism [5]–[9]. The clinical and genetic heterogeneity of autism have complicated attempts to understand its pathophysiology, especially in the majority of cases where autism is considered idiopathic. As non-syndromic autism is largely a disorder of the brain with specificity for particular brain regions and because there is no consensus animal model, studies on carefully selected postmortem human brains are needed and this, in turn, adds to the challenge of uncovering the pathophysiologic mechanisms of autism.

Despite these challenges, recent literature suggests that the initial genetic and/or environmental insults that cause autism converge on a small number of cellular pathways and processes, with data supporting multiple, non-mutually exclusive hypotheses [10]–[12]. A recent whole genome transcriptomic analysis of autism brain suggested candidate pathways in immune regulation and alternative splicing regulation in the temporal cortex, frontal cortex, and cerebellar vermis [13]. We chose to evaluate potentially disrupted pathways in autistic brain both at the gene expression and DNA methylation levels on a genome-wide scale and selected two other brain regions, cerebellar hemisphere cortex and Brodmann area 19 cortex (BA19, occipital cortex), which have been associated with autism pathogenesis based on histopathologic and neuroradiological analyses [14]–[16]. Additionally, to maximize the likelihood of determining common pathophysiologic mechanisms we chose to study brains from a subset of persons affected with autism – males with idiopathic autism - and explored how molecular heterogeneity within this group related to pathways associated with particular symptom domains.

## Materials and Methods

### Subjects and Samples

Brain tissue samples from Brodmann area 19 (BA19) occipital cortex and cerebellar hemispheric cortex were procured from subjects and controls through the Autism Tissue Program (ATP, www.atpportal.org) from the Harvard Brain Tissue Resource Center (www.brainbank.mclean.org) and the National Institute for Child Health and Human Development (NICHD) Brain and Tissue Bank (www.btbank.org). Variables selected as candidate covariates included age, gender, ethnicity, DSM-IV diagnosis of the form of autism, Autism Diagnosis Observation Schedule (ADOS) or Autism Diagnostic Interview-Revised (ADI-R) scores, Intelligence Quotient (IQ) scores, seizure history, medication history, birth history, brain neuropathology findings, cause of death, and postmortem interval where available. Inclusion criteria included: male gender; autism diagnosis by a validated psychiatric/psychologic instrument; and the availability of sufficient fresh frozen tissue available for genome-wide methylation analysis, bisulfite sequencing, and gene expression studies. Exclusion criteria included: formalin-fixation of brains, brains from individuals with a medication history of medications known or suspected to have effects on methylation (eg, valproic acid, olanzapine, and sulpiride); gross structural abnormalities of the brain; brains from individuals with a complicated birth history and/or evidence of pre- or perinatal hypoxia; history of major head trauma; diagnosis of Rett syndrome, Fragile X syndrome, tuberous sclerosis, or other syndromic process; or any known or likely pathologic cytogenetic abnormality identified by either routine karyotyping or chromosomal microarray analysis. Samples from male controls with no known developmental disorder or syndromic process were age-matched to each case. Tissues were procured from a total of 9 autism and 9 control subjects. The characteristics of the subjects are presented in Tables S1 and S2. This study was approved by the Institutional Review Board of the Cleveland Clinic and is in accord with the principles of the Declaration of Helsinki. The gene expression and DNA methylation data presented in this publication have been deposited in NCBIs Gene Expression Omnibus (GEO, http://www.ncbi.nlm.nih.gov/geo/) and are accessible through GEO Series accession numbers GSE38322 and GSE38608, respectively.

### Copy Number Evaluation

All 9 cases had high-resolution single nucleotide polymorphism (SNP) hybridization array data available from 2 platforms, the Affymetrix Genome-Wide Human SNP Array 6.0 and the Illumina Human1M-Duo DNA Analysis BeadChip. These assays were performed in the laboratory of S. Scherer, Hospital for Sick Children, Toronto [17]. We analyzed the stringent call list for each potential subject for pathological copy number variants (CNVs) by evaluating each call against the Database of Genomic Variants (DGV) (http://projects.tcag.ca/variation/) and the DECIPHER database (http://decipher.sanger.ac.uk/) as of January 2011. Subjects were included if they did not harbor CNVs that overlapped with those listed in the DECIPHER database or if they had CNVs that were not present in the DGV or DECIPHER databases but which did not contain genes with likely haploinsufficiency or strongly implicated in autism in the literature.

### Sample Processing

Total DNA and RNA were co-extracted from ∼40 mg of cerebellar cortex and ∼40 mg BA19 cortex using the Allprep DNA/RNA Kit (Qiagen) according to the manufacturer’s instructions. RNA and DNA were tested for purity and concentration on a Nanodrop device (Thermo Scientific). One microgram of genomic DNA per sample was bisulfite-converted using the EZ DNA Methylation Kit (Zymo). Samples of RNA or bisulfite-converted DNA were submitted to the Genomics Core Laboratory of the Lerner Research Institute at the Cleveland Clinic for further processing and application to either Illumina HumanRefHT12 v4 BeadChip gene expression microarrays or Illumina HumanMethylation27 BeadChip DNA methylation microarrays according to manufacturer’s instructions.

### Fragile X Syndrome Testing

All subjects had DNA analyzed for *FMR1* expanded repeats [18]. No autistic or control subjects were found to have pathologic expansions of CGG repeats in the 5′ untranslated region (Table S2).

### Statistical Analyses

Data were imported from Genome Studio (Illumina) to the R statistical environment (http://www.r-project.org/) and processed via standard Bioconductor packages: lumi, methylumi, made4, and limma (www.Bioconductor.org/packages/release/bioc/). Methylation data were color-balanced, quantile-normalized, and filtered for probes with ≤0.01 detection p-values. Expression data were quantile-normalized, variance-stabilized, and filtered from probes with ≤0.01 detection p-values. Correspondence analysis was carried out with the made4 Bioconductor package. Due to heterogeneity of expression data, we either included all samples to identify robust phenotype-associated differential expression or performed differential expression analysis on the set of 3 autistic cerebellar outliers compared with age-matched, non-outlier controls, as noted in the text. The 3 autistic cerebellar outliers were defined by visual inspection based on the correspondence analysis, and the two most extreme outlier samples were re-assessed for sampling or processing errors by re-extracting tissue RNA and evaluating the samples on new microarrays, which showed similar patterns of gene expression when compared with the original samples.

Array data for methylation were analyzed in two ways: (1) targeted candidate probes based on prior literature were compared between cases and matched controls using limma with a paired design. P-values were Bonferroni corrected to a familywise type I error rate of 5% and (2) genome-wide analysis was carried out comparing cases and controls in limma using a paired design. Top gene lists of up to 300 probes were generated using a filter of false discovery rate ≤5%. If no genes were differentially expressed or methylated, we also attempted to relax criteria to FDR ≤25%. Expression data were analyzed with a genome-wide discovery approach, controlling for brain region with FDR threshold of ≤5%. Analyses of subject characteristics and global methylation values were performed in SAS 9.2 (http://www.sas.com/) and JMP 8 (http://www.jmp.com/) software packages.

A priori power analysis pertaining to methylation data in candidate genes indicated at least 95% power to detect a change of 0.3 in beta value between autistic and control brain with a family-wise error rate of <0.05 (based on a two-tailed, two sample t test).

### WGCNA Analysis

We used Weighted Genome Co-Expression Network Analysis (WGCNA) to determine whether expression or methylation of particular gene modules was associated with any of the covariates under study and to assess potential confounding variables. WGCNA was implemented using the WGCNA Bioconductor package (http://www.genetics.ucla.edu/labs/horvath/CoexpressionNetwork/Rpackages/WGCNA/) for R. We used block-wise network construction of the full gene expression dataset (including both brain regions) with maximum block size of 5000, power threshold of 10, merge cutting height of 0.25, and minimum module size of 60. We chose the power threshold based on visual analysis of the scale-free topology fit index as a function of power. We performed similar analysis on the full DNA methylation dataset, but with a minimum module size of 40, given the smaller probe set for the whole genome methylation microarray. There were 21 gene modules generated. A matrix of gene modules and covariates was generated for each analysis within a given brain region and associations were considered significant if they achieved a Pearson correlation p-value of less than 0.01.

### Bioinformatics Analysis

For gene ontology analysis of differentially expressed genes or gene modules, we submitted the top 300 differentially expressed probes or the module probes list to the DAVID bioinformatics tool (http://david.abcc.ncifcrf.gov/). The list was compared with a background of the total set of expressed genes in our samples (detection p-value < = 0.01) and functional annotation clustering was performed. An EASE score threshold of 0.1 was used and the top 6 functional annotation clusters were reported with enrichment scores. Enrichment scores are the negative log_10_ of p-value based on Fisher’s exact test. Ontology terms related to cellular localization, gene product function, and biological pathway were included in the analysis, and clusters were manually annotated with representative terms for the set of annotation terms in a cluster. Visualization of gene networks was carried out in Ingenuity Pathway Analysis version 11904312 (www.ingenuity.com) on the top 300 differentially expressed probes from analyses of the full dataset or cerebellar outliers. Data from human nervous system or immune system cell lines and tissue was used to generate networks. Fisher’s exact test was used to test for enrichment of transcription factor association.

### Quantitative Real-time PCR Analysis

Extracted RNA was treated with DNase I (Invitrogen) according to manufacturer’s instructions. One microgram of RNA was then reverse transcribed with SuperScript III using the VILO kit (Invitrogen). cDNA was treated with RNase H (New England Biosciences) at 0.4 microliters per reaction at 37°C for 20 minutes. RT-PCR was performed on an Eppendorf Mastercycler realplex2 instrument in 25 microliter reaction volume using Quantitect SYBR green master mix (Qiagen) and primers at a final concentration of 0.8 micromolar. Primers were tested for specificity by preparing melting curves on serial dilutions of a standard and running PCR products on a 1% agarose gel (Table S8). GAPDH levels were used as an internal control and the standard curve method of quantitation was used based on pooled RNA. Comparisons were made with at least 5 samples per group using a two-tailed Wilcoxon rank sum test on the standardized and normalized expression levels of each gene between the full control and autistic groups.

### DNA Pyrosequencing

DNA samples were submitted to the University of Nebraska Medical Center Epigenomics Core Facility and processed by the following procedure. Bisulfite treatment was carried out using 1000 ng of sample genomic DNA and the EZ DNA Methylation-Direct kit (Zymo Research, Orange, CA). To perform PCR reactions, 42 ng of bisulfite-modified DNA was used as template. The PCR reactions were performed in a total volume of 25 µl for 35 cycles using Roche Diagnostic Corporation (Indianapolis, IN) FastStart Taq DNA Polymerase (1.0 U), MgCl_2_ solution (3.5 mM), dNTPs (0.2 mM), sense primer (0.24 uM), antisense primer (0.18 µM) (Table S9), with denaturation at 95°C for 30 seconds, annealing temperature for 45 seconds at annealing temperature indicated in Table S3, and extension at 72°C for 1 minute. For a positive (high methylation level) control, Roche Diagnostic Corporation (Indianapolis, IN) human lymphocyte genomic DNA was methylated using M. SssI (CpG) methylase kit (New England Biolabs, Ipswich, MA). Methylated DNA (1000 ng) was treated with sodium bisulfite as described above. Sodium bisulfite treated Roche human lymphocyte genomic DNA (1000 ng) served as a negative (low methylation level) control. All PCR products were electrophoresed on 0.8% agarose gel, stained with ethidium bromide, and visualized for appropriate and pure product using a Bio-Rad Laboratories (Hercules, CA) Gel-Doc UV illuminator before proceeding with all analyses. Methylation percentage of each CpG was determined using a Qiagen (Valencia, CA) Pyromark Q24 pyrosequencer and sequencing primer indicated in Table S9, according to manufacturer’s recommendations.

## Results and Discussion

### Characteristics of Subjects and Brain Samples

We analyzed postmortem brain tissue from a carefully selected sample of 9 males with idiopathic autism and 9 age- and gender-matched typically developing controls (Table S1, Table S2). Eight pairs of cerebella from autistic and control subjects produced high quality gene expression microarray data; 6 controls and 4 cases from BA19 also yielded high quality microarray data (Table S2). There were no significant differences between cases and controls in age, postmortem interval or cause of death (Table S1).

### Overview of Gene Expression Differences Between Autistic and Control Brains

**Figure 1:**
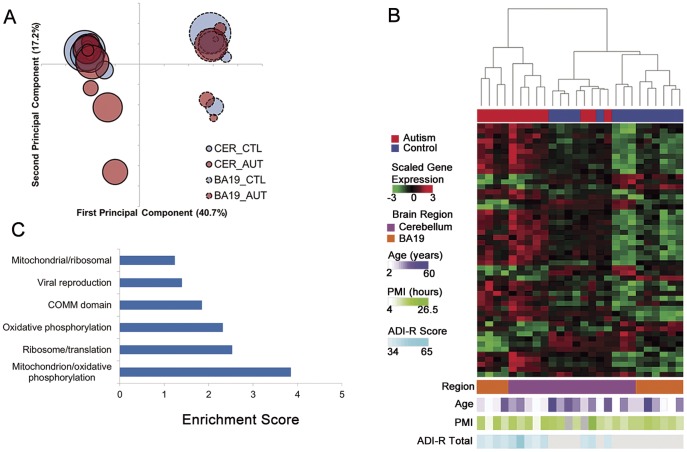
Autistic brain shows transcriptional heterogeneity and differential expression of genes of mitochondrial oxidative phosphorylation and protein translation. (**A**) Unsupervised correspondence analysis of whole-genome transcriptome expression data demonstrates that brain region is associated with the greatest source of variability in the data. The second principal component is associated with variability mostly among autistic samples. The first two principal components are listed on the x- and y-axis respectively, with percent of variance explained in parentheses. Bubbles represent individual samples, with area proportional to age, outline to brain region, and color to phenotype. (**B**) Heatmap of the top 50 differentially expressed probes between autistic and control brain, accounting for brain region, separates most autistic brains from controls (FDR ≤5%). Rows correspond to probes and columns to samples. The dendrogram represents sample similarity on the basis of the top 50 differentially expressed probes. Probes with log_2_-fold change >0.7 are listed in table S3. (**C**) The top 300 differentially expressed probes are enriched for gene ontology annotation clusters corresponding to mitochondrial oxidative phosphorylation and protein translation. ADI-R, Autism Diagnostic Interview-Revised; BA19, Brodmann area 19; CER, cerebellum; PMI, postmortem interval.

On correspondence analysis, samples segregated on the first component by brain region, as expected (Figure 1A). The second principal component separated several autistic cases and one control from the others, with heterogeneity of autistic cases most apparent in cerebella. After controlling for brain region, there were 876 unique, annotated genes differentially expressed between autistic and control brains at a false discovery rate of 5%, 32 of which had log_2_-fold changes ≥0.7 (Table S3).

Unsupervised clustering of samples by the top 50 differentially expressed probes (ranked by p-values) showed separation of most of the autism cases from controls (Figure 1B). Although there was significant transcriptional heterogeneity of the autistic brains, gene ontology enrichment analysis revealed two convergent themes: down-regulation of genes involved in mitochondrial oxidative phosphorylation and in protein translation (Figure 1C and Tables S4 and S5). This has not been reported in gene expression or protein interaction network analyses of autism [12], [19], in gene ontology analyses of large cohorts of autistic individuals with monogenic disorders and rare genomic copy number variants [20]–[24], or in recent whole-exome sequencing studies [5]–[9].

### Down-regulation of Genes of Mitochondrial Oxidative Phosphorylation and Protein Synthesis in Autistic Brain

Down-regulation of genes for mitochondrial respiratory chain complexes I, III and ATP synthase was particularly notable (Table S4). Abnormalities of mitochondrial oxidative phosphorylation are among the more commonly identified biochemical findings in individuals with autism [25], [26]. In most instances it is unclear whether these findings are primary genetic defects in oxidative phosphorylation or secondary phenomena, although some individuals having autism and mutations in genes of oxidative phosphorylation have been reported [26]. Recent work showed reduced levels of these respiratory chain complexes in autistic brain [27].

To our knowledge, down-regulation of genes of mitochondrial oxidative phosphorylation has not been reported in prior gene expression analyses of autistic brain. The differences in the results between our study and other whole genome transcriptomic analyses of autistic brain [13], [28] may be due to etiological heterogeneity together with the use of different criteria for sample selection in each study. Because of the known etiological heterogeneity of autism, we designed our study to reduce variability in the data and increase statistical power through stringent subject selection criteria (see Methods). A more heterogeneous sample in other studies could limit the ability to identify differences in the expression of mitochondrial oxidative phosphorylation-related transcripts if the findings that we observed apply only to a subset of autistic persons (eg, males with idiopathic autism). The differences in the results of our study and other transcriptomic analyses of autistic brain might also be due to differences in the regions of brain that were analyzed. It is possible that the differences in metabolic demands or brain region specific pathophysiology could account for differences in expression of mitochondrial genes. The finding of down-regulation of genes of oxidative phosphorylation, if independently confirmed, will be important in future studies of pathophysiology of autistic brain.

We also noted significant down-regulation of genes of protein synthesis. This finding suggests a generalized and non-targeted process and may be a result of dysregulation of other pathways. Alterations of neuronal protein synthesis involving selected pathways are increasingly understood to be key aspects of the pathogenesis of some forms of syndromic autism [29] and may be generalizable to many forms of cognitive and behavioral disease [30].

Our data do not inform whether the down-regulation of genes of mitochondrial oxidative phosphorylation or of protein synthesis mainly relates to glia and/or neurons. Of note, the differentially expressed genes of both mitochondrial oxidative phosphorylation and protein translation observed in this study are highly enriched for regulation by *HNF4A* in transcription factor analysis (p = 1.4E-8; Fisher’s exact test) (Figure S1). *HNF4A* is involved in tissue-specific cell differentiation and energy metabolism and has been recently identified as a cerebellar-specific marker of the hypoxia response in mouse brain [31]. To our knowledge, an involvement of *HNF4A* in the pathophysiology of autism has not been previously noted.

### Differential Expression of Genes of Synapse and Other Brain Functions

Numerous genes of brain development and function have been associated with the pathophysiology of autism with varying levels of evidence; the specific genes that are implicated vary across studies [5]–[13]. To explore specific genes that might be relevant in our dataset, we reviewed the list of probes that were differentially expressed at a log_2_-fold change of ≥0.7. Of these 32 genes, there were two genes associated with synapse/neurotransmitters (*LIN7B, SYN1*), three genes associated with vesicle transport (*VPS29, HSPB1, TUBB2B*), one gene associated with brain patterning (*GPR56*), four genes that are of significance for normal brain function based on monogenic neurologic disorders associated with mutations in those genes (*ODZ3, ATP1A2, MOCS2, PTS*) and another gene with brain-specific expression (*BEX5*) (Table S3). Eight of these genes have some evidence in support of an association with autism (http://autismkb.cbi.pku.edu.cn/) [32], two of which (*SYN1, PTS*) met thresholds for a high level of evidence [32], and concordant changes in the differential expression in autism vs control were found for three of the genes (*GPR56, HSPB1, BEX5*) between this study and the work of others [13], [28], [33]. These data provide additional support for the hypotheses that genes of synapse formation/function and of cortical development are differentially expressed in autism [20]–[24].

### Brain Immune Gene Dysregulation in a Subset of Autistic Brains

We also separately analyzed the gene expression data of the three autistic cerebellar outlier samples. Differentially expressed genes between these outliers and matched controls were enriched for NF-kB signaling and cell cycle regulation pathways (Figure S2). The top 300 differentially expressed probes were used to generate a non-redundant gene list and compared with two published differentially expressed autism gene lists in brain [13], [28] (Table S6). There was a statistically significant overlap in the lists (P = 1.4E-9; Fisher’s exact test), notably including genes associated with inflammation. While the role of neuroinflammation in autism pathophysiology remains unclear [34], our data emphasize the substantial interindividual heterogeneity of brain immune system gene dysregulation.

### Brain Gene Expression Profiles and Associations with Specific Clinical Phenotypes

**Figure 2:**
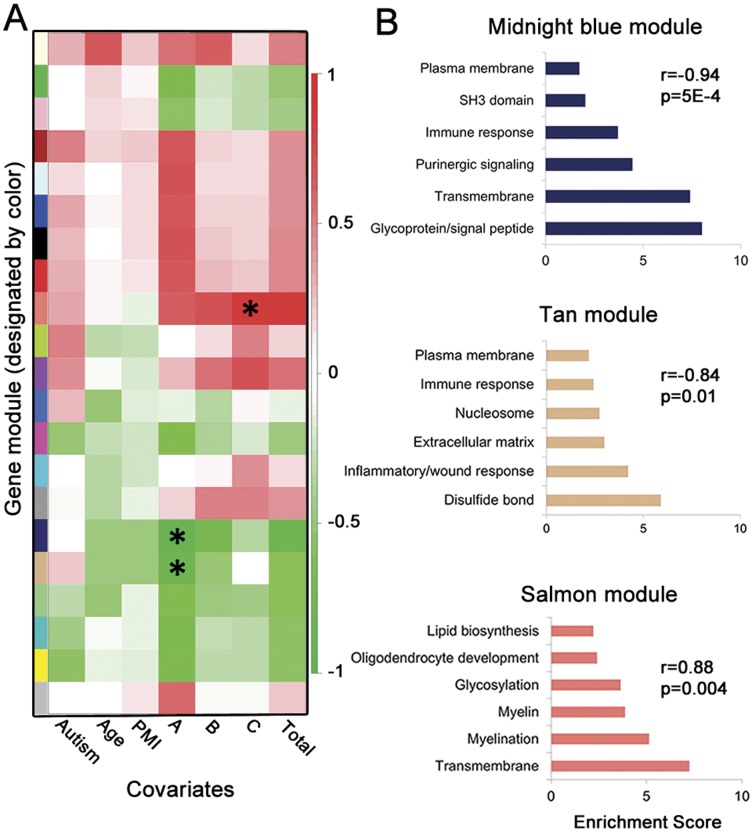
Specific domains of the Autism Diagnostic Interview-Revised (ADI-R) are associated with specific gene modules. (**A**) Heatmap of module-variable associations for cerebellar hemisphere samples from weighted genome co-expression network analysis, including domain scores for the ADI-R. Cells are color-coded by correlation coefficient and * is placed in cells having associations with p-values ≤0.01. Autism, dichotomous variable autism vs control; PMI, postmortem interval; A, ADI-R Social interaction impairments domain; B, ADI-R Communication and language impairments domain; C, ADI-R Repetitive and stereotyped behaviors domain. (**B**) Gene ontology enrichment annotation clusters for the three significant gene modules associated with specific ADI-R domain scores. P, p-value; r, Pearson correlation coefficient.

We next explored associations between phenotype and transcriptional profile with weighted genome co-expression network analysis (WGCNA) [35]. This approach can potentially identify higher order, systems level correlations. We examined potential associations between expression of all gene modules (ie, clusters of co-expressed genes) in cerebella and ADI-R total or ADI-R domain scores, which are associated with phenotypic severity in the three cardinal domains of autism: impairment of social reciprocity, impairment of verbal or non-verbal communication, and stereotyped or repetitive behaviors. Two gene modules were significantly associated with the social interaction domain of the ADI-R and one gene module significantly associated with the stereotyped and repetitive behavior domain of the ADI-R (Figure 2A). These three modules are characterized by gene ontology enrichments relating to purinergic signaling/immune response, inflammation/immune response, and myelin/myelination, respectively (Figure 2B). There were no associations for other covariates tested (Figure 2A). Correlations between autism behavioral endophenotypes and CNS gene expression patterns have not been reported.

Several types of data support the observed associations. Altered immune responses and neuroinflammation are well documented in autism [34] and there are reports of specific associations between immune parameters and deficits in social interactions and communication in autistic individuals [14]. Purinergic signaling is involved with synaptic plasticity and neuron/glia interactions [36] and recent genetic and clinical data implicates a role for purines/purinergic signaling in autism [32]. In addition, abnormalities of brain white matter are commonly observed in diffusion tensor imaging-based neuroradiological studies of autism [37] and abnormalities of cerebellar white matter have been associated with repetitive behaviors in autistic individuals [38]. Determination of the pathophysiology and clinical meanings of these associations will be important next steps.

### Validation of Gene Expression Microarray Data

Quantitative RT-PCR was used to validate the results of the microarray gene expression analyses (Figure S3). We selected candidates based on strong effect size on the gene expression microarray analyses or biological plausibility based on published data. The results of all 5 statistically significant differentially expressed transcripts by RT-PCR were concordant with microarray data, confirming the validity of transcriptomic analysis by a gene microarray approach.

### Brain Whole Genome Methylation Analysis

**Figure 3:**
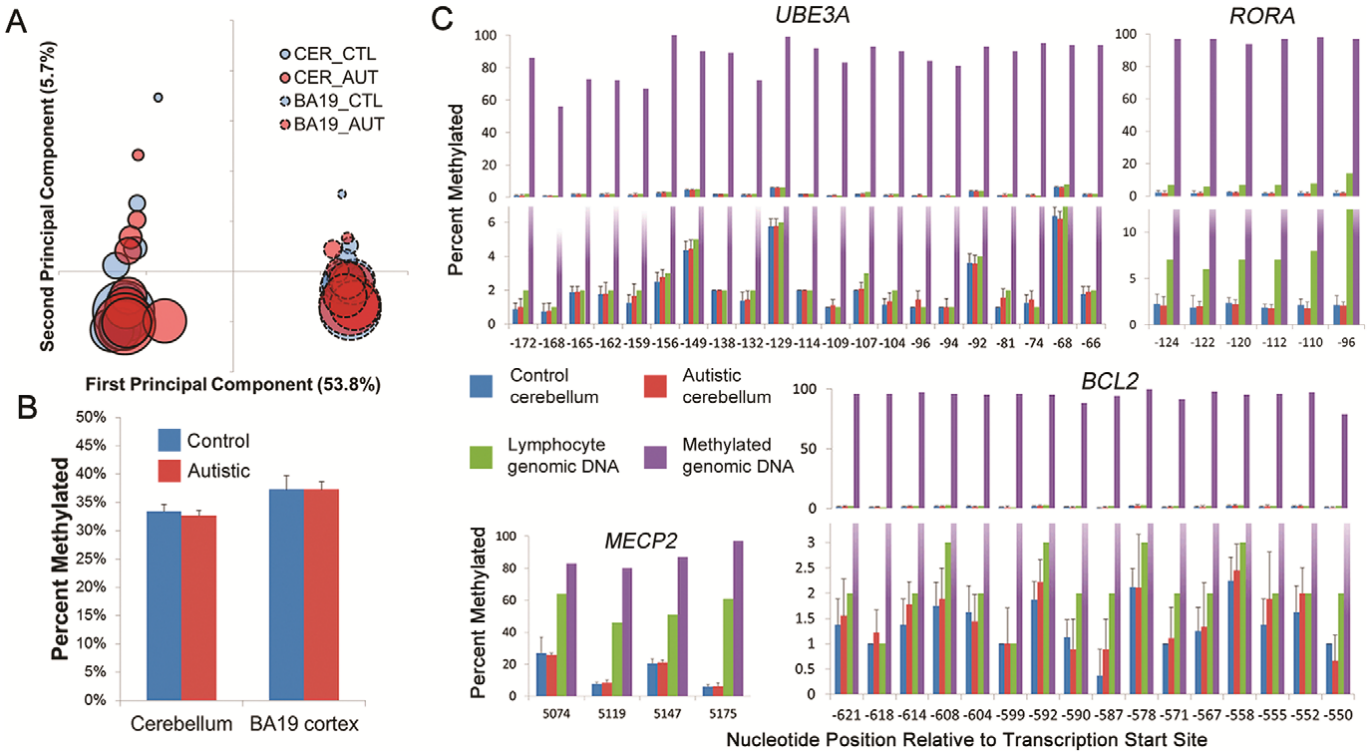
No differential methylation of genomic DNA was identified between control and autism cerebellar cortex or BA19 cortex. (**A**) Unsupervised correspondence analysis of genome-wide CpG methylation in control and autism cerebellar cortex and BA19 cerebral cortex demonstrates that brain region and age are associated with the largest two sources of variability, respectively, in the data. The first two principal components are listed on the x- and y-axis respectively, with percent of variance explained in parentheses. Bubbles correspond to individual samples with area proportional to age of subject, bubble outline to region, and color to phenotype. (**B**) Percent of genome-wide methylated probes in cerebellar cortex or BA19 cortex in control and autism samples. Error bars represent standard deviations. There was no significant difference in global methylation between autism vs control cerebellum or BA19 cortex. There was a significant difference between brain regions, as expected (alpha = 0.05; Wilcoxon rank-sum test). (**C**) Pyrosequencing of bisulfite-treated DNA. Percent methylation at individual CpG dinucleotides is reported in cerebellar cortex samples (n ≥7 per group) for *a priori* candidate genes suspected to exhibit differential methylation. X-axis represents locus relative to transcription start site. Error bars represent standard deviations.

To explore a possible epigenetic basis of the gene expression findings, we obtained high quality methylation data for 9 pairs of age- and gender-matched cerebella and 8 pairs of BA19 cortex and analyzed >27,000 probes for differential methylation at individual CpG dinucleotides enriched in promoter regions of genes. Correspondence analysis indicated that region and age were the major sources of variability in the data (Figure 3A) and, after controlling for these covariates, there were no significantly differentially methylated genes in either autistic cerebellar or BA19 cortex (data not shown). We also examined the genomic proportion of methylated loci in cerebellar and BA19 cortex. There was no significant difference between autism cases and controls within each region, but there was a statistically significant difference of the proportion of global DNA methylation between cerebellar and BA19 cortex, consistent with previous analyses of normal brain [39] (Figure 3B). We then used WGCNA to analyze the full DNA methylation dataset; there were no modules associated with the autistic phenotype in either cerebellar or BA19 cortex (data not shown).

### Methylation Analyses of Candidate Autism Genes

Several investigators previously reported epigenetic alterations in *RORA* and *BCL2* in lymphoblasts [40] or *UBE3A*, *MECP2*, and *OXTR* in brain tissue [41]–[43] from small numbers of individuals with autism. We evaluated each of these genes using the DNA methylation microarray data and did not find differential methylation for any of the multiple probes associated with each of the genes (Table S9). Although CpG dinucleotides within a particular promoter CpG island are generally correlated with each other and with gene regulation, we could not exclude the possibility that some unmeasured CpG dinucleotides within an island we investigated were differentially methylated. We therefore performed bisulfite pyrosequencing of the most relevant regions of the CpG islands; no differentially methylated CpG dinucleotides were noted in *MECP2*, *UBE3A*, *BCL2*, *RORA*, *OXTR* or *CEBPD* (Figure 3C, Figure S4, Table S7).

### Strengths and Limitations of the Current Work

We recognize several limitations to our study. First, the sample size is necessarily relatively small as it is difficult to procure brain specimens from numerous persons with autism, particularly after stringent selection criteria are used. Second, as is the case for any study, we cannot exclude that our results are artifacts of an unmeasured covariate. However, we tried to exclude potential confounders by exploring postmortem interval, cause of death, seizure comorbidity and age, as well as tissue variables such as RNA quality and tissue sampling error. Third, although we had excellent genomic coverage on both our methylation and gene expression arrays, we did not have complete coverage and we may have missed small epigenomic or transcriptional changes with pathologic relevance. We did not evaluate histone modifications, which can account for expression differences in the absence of differential DNA methylation. Fourth, we evaluated two carefully chosen brain regions but in so doing may have missed important gene expression or DNA methylation changes in other brain regions. Fifth, we evaluated whole tissue extracts and might have missed epigenetic or transcriptional dysregulation specific to a subpopulation of clinically relevant cells.

This study has several strengths. More than 100 uncommon or rare causes of autism are currently described yet autistic individuals with defined syndromic, metabolic or chromosomal etiologies comprise a minority of all individuals with autism. The pathophysiologic processes operative in some, or perhaps most, persons with these uncommon causes of autism may not necessarily be the same as the disease processes present in persons with idiopathic autism. Our subject selection process was, therefore, rigorous and included evaluation of clinical and molecular biologic data to produce a set of stringently defined idiopathic autistic male brains. We used high resolution microarrays to provide a detailed map of the epigenetic and transcriptional profiles of the analyzed brain regions. We also used complementary confirmatory techniques to evaluate candidate genes.

### Conclusions

Taken together, our analyses demonstrate significant transcriptional heterogeneity in autistic brains with decreased expression of mitochondrial oxidative phosphorylation and protein synthesis-related genes. The latter suggests that convergence of pathobiology in autism may occur at the level of fundamental cell processes such as mitochondrial energy metabolism and protein translation. Nonetheless, the transcriptional heterogeneity noted here also raises the possibility there may be diverse sets of biological pathways that are also dysregulated in different autistic individuals and this, in turn, may be relevant in subtyping individuals for clinical trials. Additionally, our data suggest that there are particular biological pathways associated with different symptom domains in autism. Perhaps clinical heterogeneity in autism is the result of differences in these specific pathways which act to control domain severities semi-independently. We found that DNA methylation of gene promoters is not widely disrupted in the autistic cerebellum or BA19 brain regions, suggesting that transcriptional changes are largely the result of other regulatory mechanisms.

## Supporting Information

Figure S1 **Top genes differentially expressed between autism and control brain accounting for region are enriched for regulation by HNF4A.** Genes colored green are down-regulated and those colored red are up-regulated. Color intensity is proportional to log2-fold change between autistic and control groups. Transcription factor enrichment was carried out using Ingenuity Pathway Analysis transcription factor analysis, which showed a strong enrichment for HNF4A (p = 1.4E-8; Fisher’s exact test).
(DOCM)

Figure S2 **Pathways enriched for differentially expressed genes in autistic outliers.** A. The top gene network for differentially expressed genes in cerebellar outlier samples vs. non-outlier control samples is associated with NF-κB signaling. B. The second gene network for differentially expressed genes in cerebellar outlier samples vs. non-outlier controls is associated with cell cycle regulation. Gene network analysis was carried out using Ingenuity Pathway Analysis.
(DOCM)

Figure S3 **Results of reverse transcriptase real-time quantitative PCR.** A. GAPDH-normalized fold changes of autistic vs. control cerebellar cortex gene expression for genes differentially expressed on microarrays. Direction of expression change on the microarray and in rtPCR assays is shown with p-values for Wilcoxon rank sum test. B. GAPDH-normalized fold changes of autistic vs. control BA19 cortex gene expression for candidate genes from literature or cerebellar cortex results. C. GAPDH-normalized fold changes of autistic vs. control cerebellar cortex gene expression in candidate genes from literature. Significant findings at a threshold value of 0.05 are denoted with an asterisk in the figure. Data were obtained from all cerebellar samples and at least 5 BA19 samples per group.
(DOCM)

Figure S4 **Pyrosequencing of bisulfite-converted DNA in CpG islands for OXTR and CEBPD.** Percent methylation at individual CpG dinucleotides is reported in cerebellar cortex samples (n ≥7 per group). X-axis represents locus relative to transcription start site. Human lymphocyte genomic DNA and enzymatically methylated human lymphocyte genomic DNA were used as controls. Error bars represent standard deviations.
(DOCM)

Table S1 **Characteristics of study subjects.** All subjects were male and cases were matched to controls for age within one year. †All other ethnicity values were “white”.
(DOC)

Table S2 **Individual subject characteristics.** Ethnicity was obtained from ATP database when available. COD, cause of death; A, Social interaction impairments domain; B, Communication and language impairments domain; C, Repetitive and stereotyped behaviors domain; D = Symptom onset before 36 months of age; ADI-R total = sum of scales A through D. All subjects were represented in DNA methylation experiments. FMR1 repeat length refers to the number of CGG trinucleotide repeats in the 5′ untranslated region of the FMR1 gene.
(DOC)

Table S3 **Top differentially expressed probes between autistic and control brain after controlling for brain region.** The top differentially expressed genes between autistic and control brain are listed at a FDR <5% and a log2-fold change of >0.7. P-values were adjusted by the method of Benjamini and Hochberg. OMIM mendelian disorders were listed if applicable. Gene description was from Illumina annotation. FC, fold change; OMIM, Online Mendelian Inheritance in Man.
(DOC)

Table S4 **Probes involved in oxidative phosphorylation differentially expressed between autistic and control brain after controlling for brain region.** The top 300 differentially expressed probes between autistic and control brain at a FDR <5% included 17 oxidative phosphorylation genes identified in Ingenuity Pathway Analysis (pathway enrichment for oxidative phosphorylation p = 5.8E-14; Fisher’s exact test). P-values were adjusted by the method of Benjamini and Hochberg. OMIM mendelian disorders were listed if applicable. FC, fold change; OMIM, Online Mendelian Inheritance in Man.
(DOC)

Table S5 **Probes associated with ribosomes differentially expressed between autistic and control brain after controlling for brain region.** The top 300 differentially expressed probes between autistic and control brain at a FDR <5% included 12 ribosomal/protein translation genes identified in Ingenuity Pathway Analysis (pathway enrichment for EIF2 signaling p = 1.0E-6; Fisher’s exact test). P-values were adjusted by the method of Benjamini and Hochberg. OMIM mendelian disorders were listed if applicable. FC, fold change; OMIM, Online Mendelian Inheritance in Man.
(DOC)

Table S6 **Differentially expressed genes in autism outlier cerebellar vs. age-matched controls.** The top 300 DE probes at a FDR of <5% were consolidated into a non-redundant, mappable list of 75 genes. Log2-fold change in autism is reported for our dataset. There were six directly overlapping DE genes from Voineagu et al. and Garbett et al. top gene lists from temporal cortex, which was statistically significant (p = 1.4E-9, OR = 67 [95%CI: 23–170]; Fisher’s exact test).
(DOC)

Table S7 **Previously implicated genes for DNA methylation abnormalities were not differentially methylated between autistic and control BA19 or cerebellar cortex by microarray analysis.** Probes for BCL2, MECP2, OXTR, RORA, and UBE3A were compared between 9 matched autistic and control BA19 or cerebellar cortical samples. P-values are for tests analogous to two-tailed, paired t-tests. Differences are for group mean M-values (M-values are logit transformed beta-values, where beta-value is approximately the proportion of methylation at a particular locus). No probes were statistically significant, controlling for multiple comparisons (adjusted p-value = 0.002). All nucleotide positions are for the NCBI36/hg18 genome build.
(DOC)

Table S8 **Primers used for real-time reverse-transcriptase quantitative PCR.** Primers were designed to span introns or lie on an exon-exon junction. See Methods for description of assay conditions.
(DOCX)

Table S9 **Primers used for pyrosequencing of bisulfite-converted DNA.**
(DOC)
