# Supplementary material for: Brain Transcriptional and Epigenetic Associations with Autism
Source: PLoS One. 2012 Sep 12;7(9):e44736. doi: 10.1371/journal.pone.0044736 (PMC3440365; doi:10.1371/journal.pone.0044736)
Supplement: Figure S1 — Top genes differentially expressed between autism and control brain accounting for region are enriched for regulation by HNF4A. Genes colored green are down-regulated and those colored red are up-regulated. Color intensity is proportional to log2-fold change between autistic and control groups. Transcription factor enrichment was carried out using Ingenuity Pathway Analysis transcription factor analysis, which showed a strong enrichment for HNF4A (p = 1.4E-8; Fisher’s exact test). (DOCM) [file pone.0044736.s001.doc]

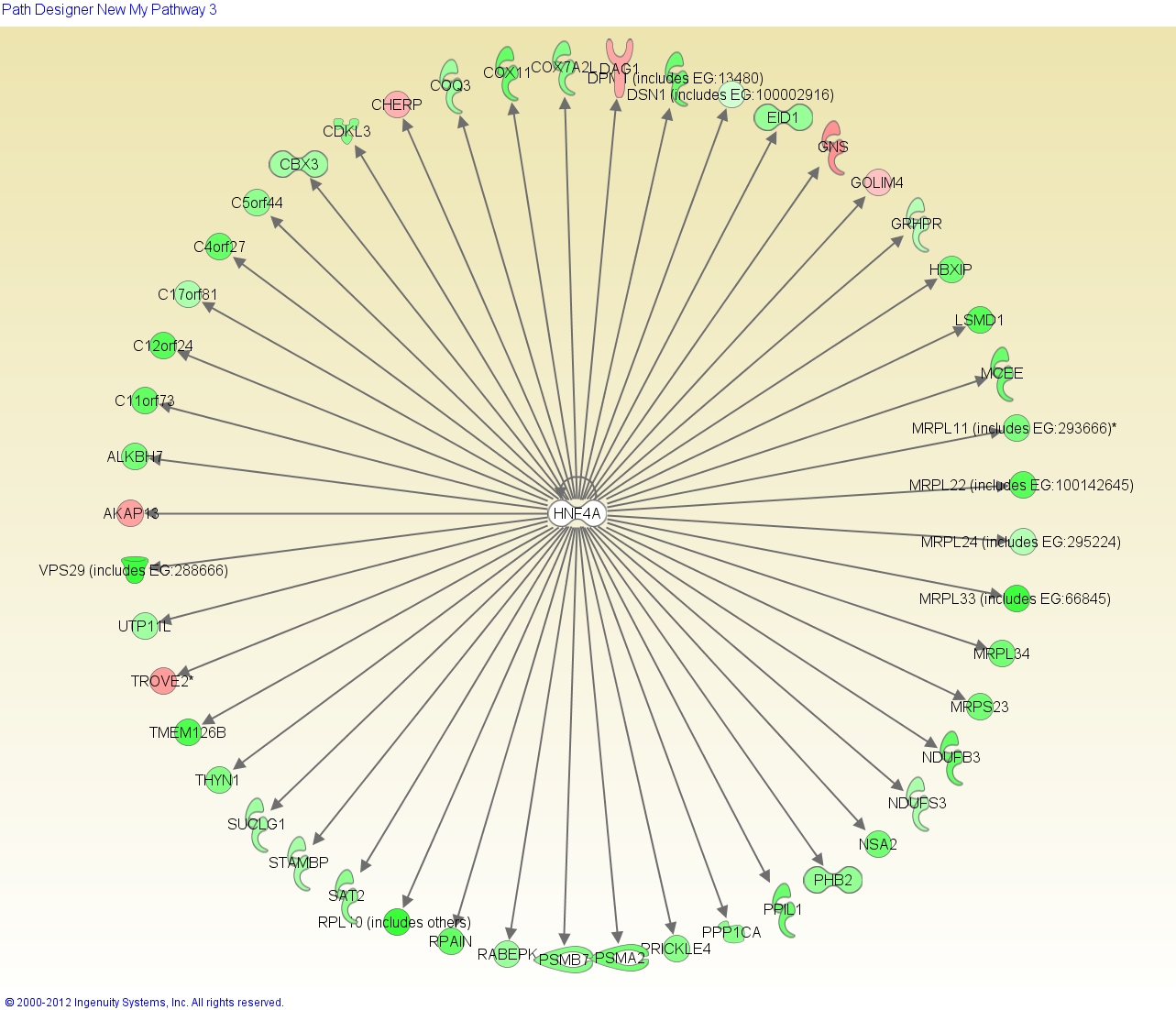


**Fig. S1. Top genes differentially expressed between autism and control brain accounting for region are enriched for regulation by *HNF4A.*** Genes colored green are down-regulated and those colored red are up-regulated. Color intensity is proportional to log_2_-fold change between autistic and control groups. Transcription factor enrichment was carried out using Ingenuity Pathway Analysis transcription factor analysis, which showed a strong enrichment for *HNF4A* (p=1.4E-8; Fisher’s exact test).
