# Supplementary material for: Brain Transcriptional and Epigenetic Associations with Autism
Source: PLoS One. 2012 Sep 12;7(9):e44736. doi: 10.1371/journal.pone.0044736 (PMC3440365; doi:10.1371/journal.pone.0044736)
Supplement: Figure S2 — Pathways enriched for differentially expressed genes in autistic outliers. A. The top gene network for differentially expressed genes in cerebellar outlier samples vs. non-outlier control samples is associated with NF-κB signaling. B. The second gene network for differentially expressed genes in cerebellar outlier samples vs. non-outlier controls is associated with cell cycle regulation. Gene network analysis was carried out using Ingenuity Pathway Analysis. (DOCM) [file pone.0044736.s002.doc]

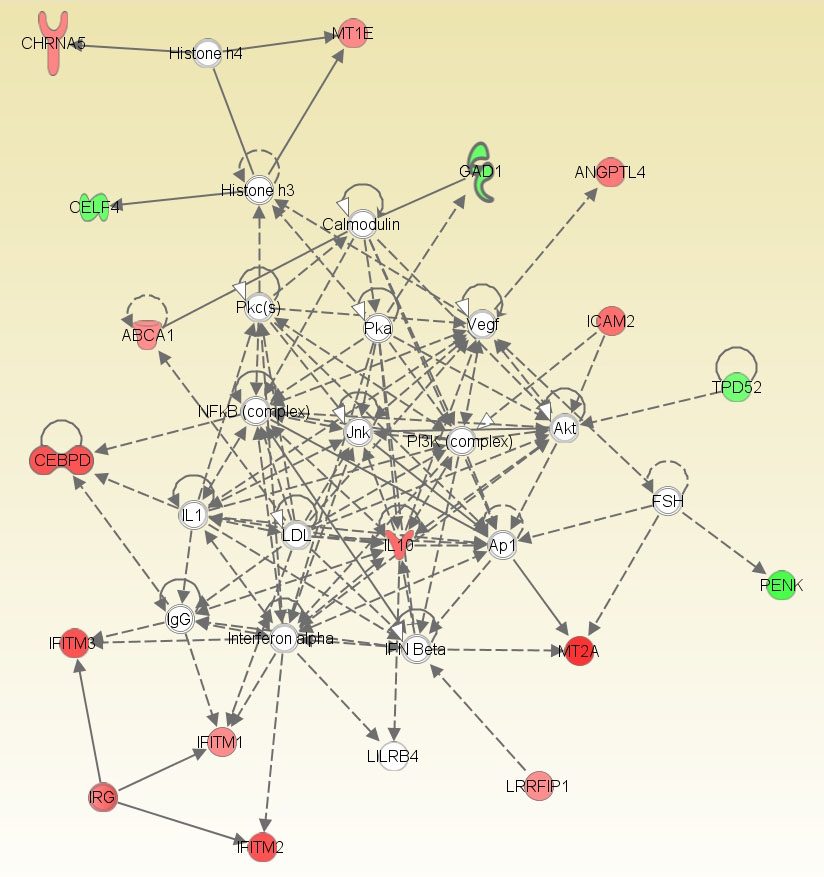

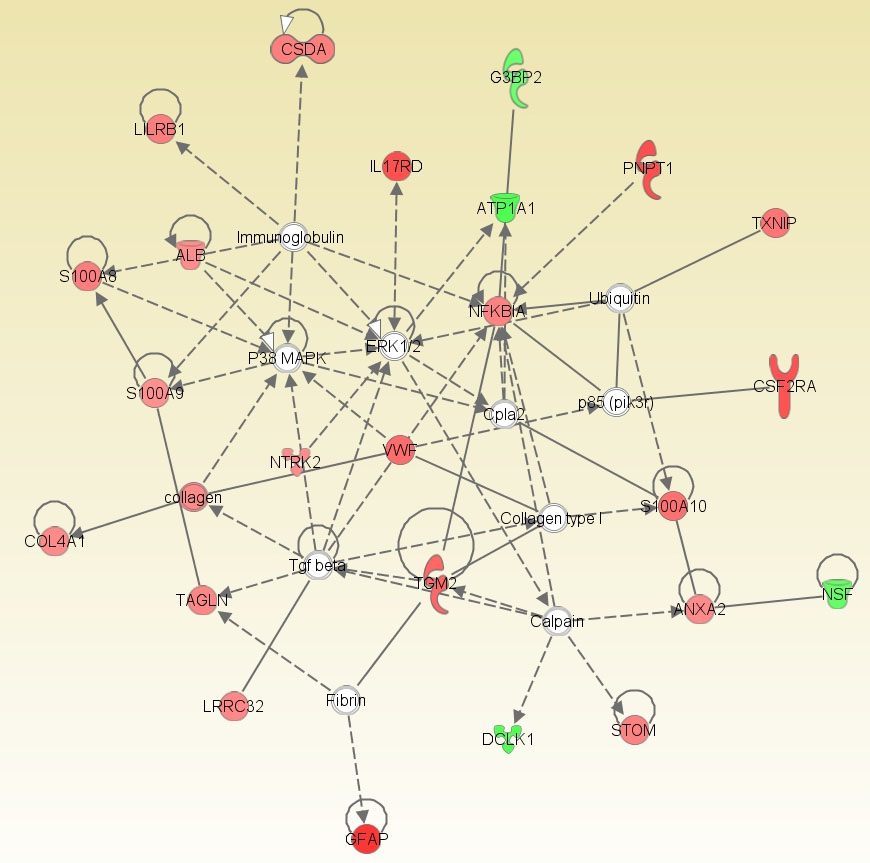


**B**

**Aa**

**Fig. S2. Pathways enriched for differentially expressed genes in autistic outliers.** **A**. The top gene network for differentially expressed genes in cerebellar outlier samples vs. non-outlier control samples is associated with NF-κB signaling. **B**. The second gene network for differentially expressed genes in cerebellar outlier samples vs. non-outlier controls is associated with cell cycle regulation. Gene network analysis was carried out using Ingenuity Pathway Analysis.
