# Supplementary material for: Brain Transcriptional and Epigenetic Associations with Autism
Source: PLoS One. 2012 Sep 12;7(9):e44736. doi: 10.1371/journal.pone.0044736 (PMC3440365; doi:10.1371/journal.pone.0044736)
Supplement: Figure S3 — Results of reverse transcriptase real-time quantitative PCR. A. GAPDH-normalized fold changes of autistic vs. control cerebellar cortex gene expression for genes differentially expressed on microarrays. Direction of expression change on the microarray and in rtPCR assays is shown with p-values for Wilcoxon rank sum test. B. GAPDH-normalized fold changes of autistic vs. control BA19 cortex gene expression for candidate genes from literature or cerebellar cortex results. C. GAPDH-normalized fold changes of autistic vs. control cerebellar cortex gene expression in candidate genes from literature. Significant findings at a threshold value of 0.05 are denoted with an asterisk in the figure. Data were obtained from all cerebellar samples and at least 5 BA19 samples per group. (DOCM) [file pone.0044736.s003.doc]

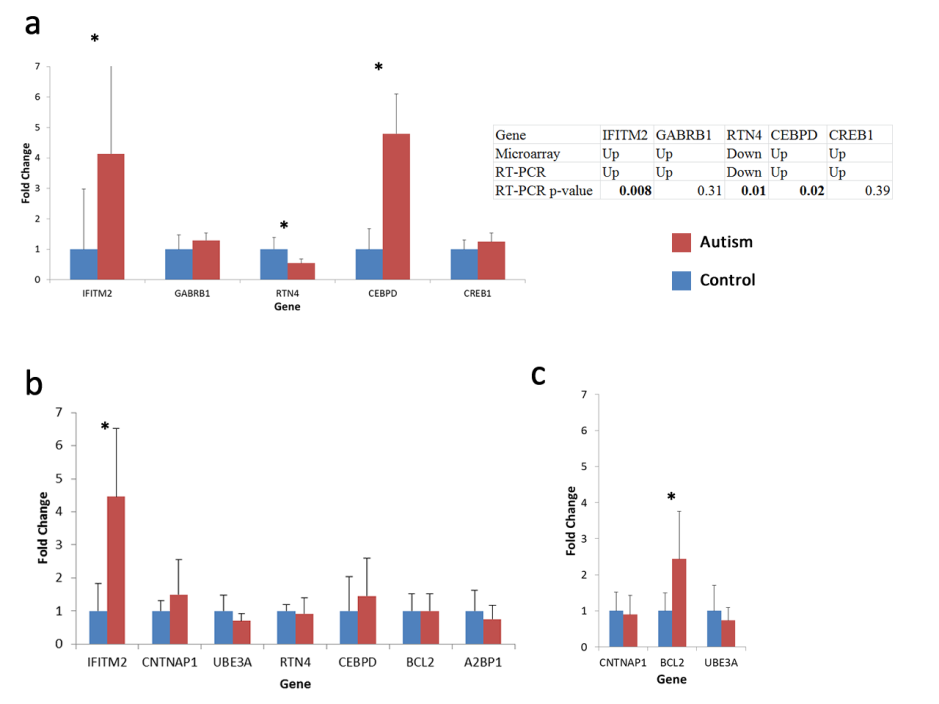


**Fig. S3. Results of reverse transcriptase real-time quantitative PCR.** **A.** GAPDH-normalized fold changes of autistic vs. control cerebellar cortex gene expression for genes differentially expressed on microarrays. Direction of expression change on the microarray and in rtPCR assays is shown with p-values for Wilcoxon rank sum test. **B.** GAPDH-normalized fold changes of autistic vs. control BA19 cortex gene expression for candidate genes from literature or cerebellar cortex results. **C.** GAPDH-normalized fold changes of autistic vs. control cerebellar cortex gene expression in candidate genes from literature. Significant findings at a threshold value of 0.05 are denoted with an asterisk in the figure. Data were obtained from all cerebellar samples and at least 5 BA19 samples per group.
