# Supplementary material for: Brain Transcriptional and Epigenetic Associations with Autism
Source: PLoS One. 2012 Sep 12;7(9):e44736. doi: 10.1371/journal.pone.0044736 (PMC3440365; doi:10.1371/journal.pone.0044736)
Supplement: Figure S4 — Pyrosequencing of bisulfite-converted DNA in CpG islands for OXTR and CEBPD. Percent methylation at individual CpG dinucleotides is reported in cerebellar cortex samples (n ≥7 per group). X-axis represents locus relative to transcription start site. Human lymphocyte genomic DNA and enzymatically methylated human lymphocyte genomic DNA were used as controls. Error bars represent standard deviations. (DOCM) [file pone.0044736.s004.doc]

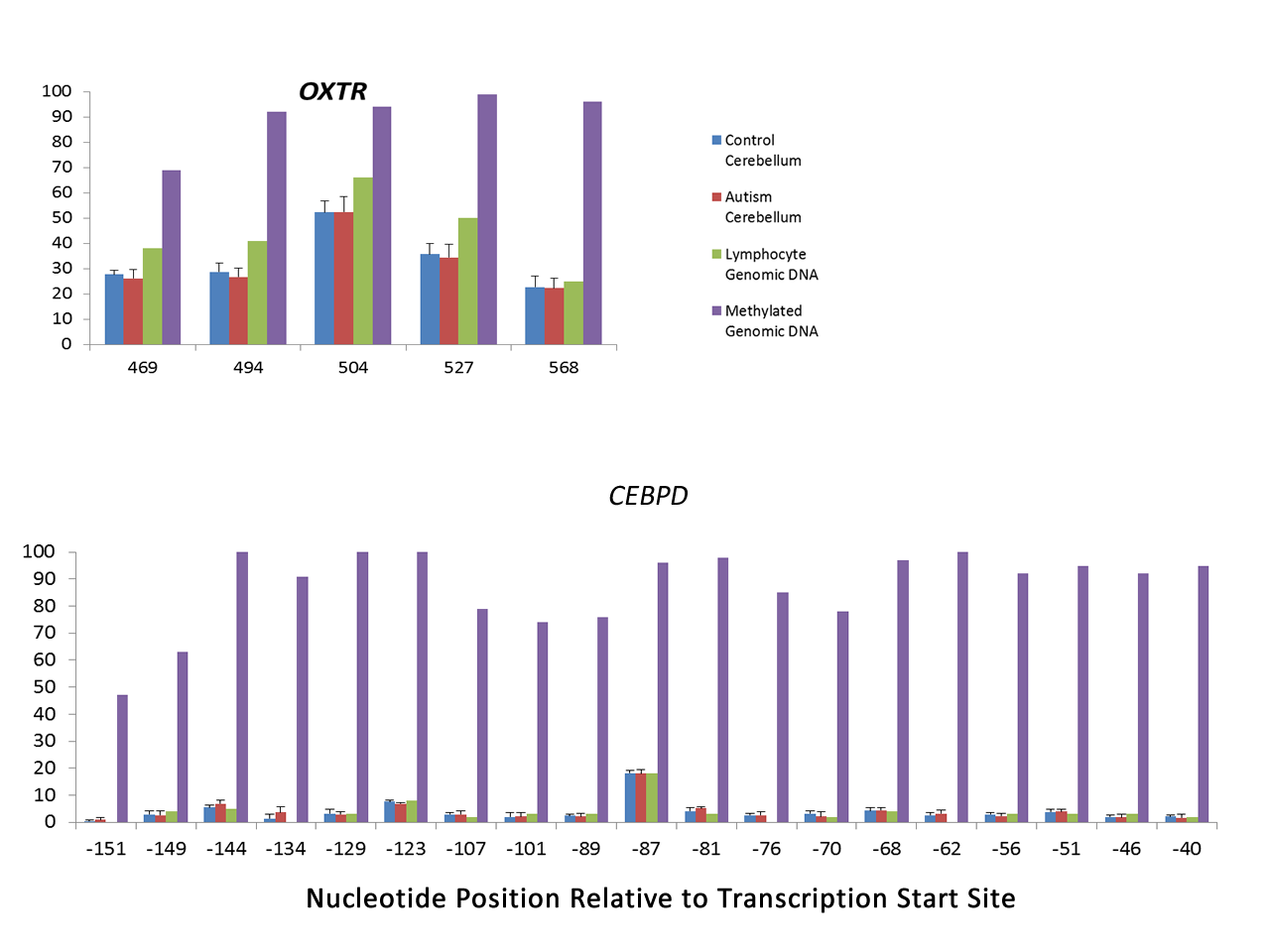


**Fig. S4. Pyrosequencing of bisulfite-converted DNA in CpG islands for OXTR and CEBPD.** Percent methylation at individual CpG dinucleotides is reported in cerebellar cortex samples (n ≥7 per group). X-axis represents locus relative to transcription start site. Human lymphocyte genomic DNA and enzymatically methylated human lymphocyte genomic DNA were used as controls. Error bars represent standard deviations.
