# Supplementary material for: Brain Transcriptional and Epigenetic Associations with Autism
Source: PLoS One. 2012 Sep 12;7(9):e44736. doi: 10.1371/journal.pone.0044736 (PMC3440365; doi:10.1371/journal.pone.0044736)
Supplement: Table S1 — Characteristics of study subjects. All subjects were male and cases were matched to controls for age within one year. †All other ethnicity values were “white”. (DOC) [file pone.0044736.s005.doc]

**Table S1. Characteristics of study subjects.**

| **Attribute** | **Cases (n=9)** | **Controls (n=9)** |
| --- | --- | --- |
| **Median age (range)** | 22 (2-60) | 22 (1-60) |
| **Median postmortem interval in hours (inter-quartile range)** | 23.6 (14.5-26.3) | 18.5 (13.5-21.1) |
| **% Unknown ethnicity†** | 22 | 56 |
| **Cause of death (n)** | Suffocation (2), cancer (2), sudden cardiovascular (1), other (3), unknown (1) | Suffocation (3), sudden cardiovascular (4), other (1), unknown (1) |

All subjects were male and cases were matched to controls for age within one year. †All other ethnicity values were “white”.
