# Supplementary material for: Brain Transcriptional and Epigenetic Associations with Autism
Source: PLoS One. 2012 Sep 12;7(9):e44736. doi: 10.1371/journal.pone.0044736 (PMC3440365; doi:10.1371/journal.pone.0044736)
Supplement: Table S2 — Individual subject characteristics. Ethnicity was obtained from ATP database when available. COD, cause of death; A, Social interaction impairments domain; B, Communication and language impairments domain; C, Repetitive and stereotyped behaviors domain; D = Symptom onset before 36 months of age; ADI-R total = sum of scales A through D. All subjects were represented in DNA methylation experiments. FMR1 repeat length refers to the number of CGG trinucleotide repeats in the 5′ untranslated region of the FMR1 gene. (DOC) [file pone.0044736.s006.doc]

**Table S2. Individual subject characteristics.**

| **Subject ATP ID:** | **Diagnosis** | **Regions** | **Age (years)** | **PMI (hours)** | **COD** | **Ethnicity** | **Medications** | **Seizures** | **A** | **B** | **C** | **D** | **ADI-R Total** | **Gene Expression Data (Y/N)** | ***FMR1* Repeat Length** |
| --- | --- | --- | --- | --- | --- | --- | --- | --- | --- | --- | --- | --- | --- | --- | --- |
| **AN03345** | Autism | Cerebellum, BA19 | 2 | 4 | Hypoxia | White | None reported | No/not reported | 14 | 9 | 6 | 5 | 34 | Y | 23 |
| **AN17678** | Autism | Cerebellum | 11 |  | Other | White | None reported | No/not reported | 22 | 12 | 4 | 5 | 43 | Y | 31 |
| **AN11989** | Autism | Cerebellum, BA19 | 30 | 16 | Cardiac | White | None reported | No/not reported | 26 | 22 | 12 | 5 | 65 | Y | 37 |
| **AN09730** | Autism | Cerebellum, BA19 | 22 | 25 | Hypoxia | White | Aripiprazole, lamotrigine, fish oil, multivitamin, zonisamide | Yes | 28 | 14 | 6 | 5 | 53 | Y | 30 |
| **AN08166** | Autism | Cerebellum, BA19 | 28 | 43 | Other | Unknown | Ziprasidone, cambamazepine, fexofenadine | Yes | 22 | 16 | 5 | 3 | 46 | Y | 20 |
| **AN19511** | Autism | Cerebellum, BA19 | 8 | 22.2 | Cancer | White | None reported | Yes | 19 | 14 | 4 | 2 | 39 | Y | 20 |
| **AN06420** | Autism | Cerebellum, BA19 | 39 | 14 | Cardiac | White | None reported | No/not reported | 20 | 12 | 4 | 5 | 41 | Y | 32 |
| **AN08873** | Autism | Cerebellum, BA19 | 5 | 25.5 | Hypoxia | White | Fluoxetine | No/not reported | 22 | 14 | 6 | 5 | 47 | Y | 30 |
| **AN09714** | Autism | Cerebellum, BA19 | 60 | 26.5 | Cancer | Unknown | Omeprazole, docusate, diazepam, phenobarbital, carbamazepine, guaifenesin | Yes | NA | NA | NA | NA | NA | N | 32 |
| **AN15622** | Control | Cerebellum, BA19 | 30 | 15 | Hypoxia | White | None reported | No/not reported | |  |  |  |  | Y | 23 |
| **AN10723** | Control | Cerebellum, BA19 | 60 | 24.2 | Unknown | Unknown | None reported | No/not reported | |  |  |  |  | Y | 39 |
| **AN05475** | Control | Cerebellum, BA19 | 39 |  | Cardiac | Unknown | None reported | No/not reported | |  |  |  |  | Y | 30 |
| **AN10833** | Control | Cerebellum, BA19 | 22 | 21.5 | Unknown | Unknown | None reported | No/not reported | |  |  |  |  | Y | 23 |
| **UMB4670** | Control | Cerebellum, BA19 | 4 | 17 | Cardiac | White | None reported | No/not reported | |  |  |  |  | Y | 30 |
| **BTB1453** | Control | Cerebellum, BA19 | 1 | 19 | Unknown | Black | None reported | No/not reported | |  |  |  |  | Y | 30 |
| **BTB3228** | Control | Cerebellum | 11 | 20 | Other | Unknown | None reported | No/not reported | |  |  |  |  | Y | 39 |
| **UMB4543** | Control | Cerebellum, BA19 | 28 | 13 | Other | White | None reported | No/not reported | |  |  |  |  | Y | 30 |
| **UMB1860** | Control | Cerebellum, BA19 | 8 | 5 | Cardiac | White | None reported | No/not reported | |  |  |  |  | N | 23 |

Ethnicity was obtained from ATP database when available. COD, cause of death; A, Social interaction impairments domain; B, Communication and language impairments domain; C, Repetitive and stereotyped behaviors domain; D=Symptom onset before 36 months of age; ADI-R total=sum of scales A through D. All subjects were represented in DNA methylation experiments. *FMR1* repeat length refers to the number of CGG trinucleotide repeats in the 5’ untranslated region of the *FMR1* gene.
