# Supplementary material for: Brain Transcriptional and Epigenetic Associations with Autism
Source: PLoS One. 2012 Sep 12;7(9):e44736. doi: 10.1371/journal.pone.0044736 (PMC3440365; doi:10.1371/journal.pone.0044736)
Supplement: Table S3 — Top differentially expressed probes between autistic and control brain after controlling for brain region. The top differentially expressed genes between autistic and control brain are listed at a FDR <5% and a log2-fold change of >0.7. P-values were adjusted by the method of Benjamini and Hochberg. OMIM mendelian disorders were listed if applicable. Gene description was from Illumina annotation. FC, fold change; OMIM, Online Mendelian Inheritance in Man. (DOC) [file pone.0044736.s007.doc]

**Table S3. Top differentially expressed probes between autistic and control brain after controlling for brain region.**

| **Gene symbol** | **Illumina probe ID** | **Log_2_ -FC** | **P-value** | **Adjusted p-value** | **Probe description** | **OMIM disorder** |
| --- | --- | --- | --- | --- | --- | --- |
| **MRPL54** | ILMN_1658486 | -0.83 | 9.59E-07 | 0.009 | Homo sapiens mitochondrial ribosomal protein L54 (MRPL54), nuclear gene encoding mitochondrial protein, mRNA. | |
| **ATP5L** | ILMN_2079285 | -0.73 | 8.03E-06 | 0.020 | Homo sapiens ATP synthase, H+ transporting, mitochondrial F0 complex, subunit G (ATP5L), nuclear gene encoding mitochondrial protein, mRNA. | |
| **LIN7B** | ILMN_1663444 | -0.80 | 9.00E-06 | 0.020 | Homo sapiens lin-7 homolog B (C. elegans) (LIN7B), mRNA. | |
| **MRPL33** | ILMN_1726417 | -0.72 | 2.91E-05 | 0.022 | Homo sapiens mitochondrial ribosomal protein L33 (MRPL33), nuclear gene encoding mitochondrial protein, transcript variant 1, mRNA. | |
| **C20orf7** | ILMN_1813344 | -0.70 | 3.37E-05 | 0.022 | Homo sapiens chromosome 20 open reading frame 7 (C20orf7), transcript variant 1, mRNA. | |
| **LOC389342** | ILMN_3280565 | -0.74 | 4.14E-05 | 0.022 | PREDICTED: Homo sapiens similar to QM protein, transcript variant 11 (LOC389342), mRNA. | |
| **RIPPLY2** | ILMN_2200915 | -0.78 | 5.70E-05 | 0.023 | Homo sapiens ripply2 homolog (zebrafish) (RIPPLY2), mRNA. | |
| **RPL8** | ILMN_1811433 | -0.73 | 5.89E-05 | 0.023 | Homo sapiens ribosomal protein L8 (RPL8), transcript variant 2, mRNA. | |
| **ODZ3** | ILMN_2336094 | 0.74 | 6.37E-05 | 0.023 | Homo sapiens odz, odd Oz/ten-m homolog 3 (Drosophila) (ODZ3), mRNA. | Spinocerebellar ataxia 30 (%613371) |
| **GPR56** | ILMN_2352097 | 0.90 | 7.15E-05 | 0.023 | Homo sapiens G protein-coupled receptor 56 (GPR56), transcript variant 2, mRNA. | Bilateral frontoparietal polymicrogyria (#606854) |
| **GPR56** | ILMN_2384122 | 0.97 | 7.41E-05 | 0.023 | Homo sapiens G protein-coupled receptor 56 (GPR56), transcript variant 3, mRNA. | Bilateral frontoparietal polymicrogyria (#606854) |
| **C20orf7** | ILMN_2350607 | -0.70 | 7.47E-05 | 0.023 | Homo sapiens chromosome 20 open reading frame 7 (C20orf7), transcript variant 1, mRNA. | |
| **TMEM14B** | ILMN_1685258 | -0.73 | 8.33E-05 | 0.024 | Homo sapiens transmembrane protein 14B (TMEM14B), mRNA. | |
| **TCTEX1D2** | ILMN_1700955 | -0.76 | 1.04E-04 | 0.025 | Homo sapiens Tctex1 domain containing 2 (TCTEX1D2), mRNA. | |
| **TMEM14D** | ILMN_3244248 | -0.76 | 1.18E-04 | 0.025 | PREDICTED: Homo sapiens transmembrane protein 14D (TMEM14D), mRNA. | |
| **RPL10A** | ILMN_1808041 | -0.72 | 1.32E-04 | 0.025 | Homo sapiens ribosomal protein L10a (RPL10A), mRNA. | |
| **ATP1A2** | ILMN_1777411 | 0.73 | 1.32E-04 | 0.025 | Homo sapiens ATPase, Na+/K+ transporting, alpha 2 (+) polypeptide (ATP1A2), mRNA. | Familial hemiplegic migraine (#602481) |
| **YEATS4** | ILMN_1801387 | -0.89 | 1.57E-04 | 0.025 | Homo sapiens YEATS domain containing 4 (YEATS4), mRNA. | |
| **PTS** | ILMN_1720322 | -0.75 | 1.68E-04 | 0.025 | Homo sapiens 6-pyruvoyltetrahydropterin synthase (PTS), mRNA. | Hyperphenylalaninemia, BH4-deficient (#261640) |
| **LOC729769** | ILMN_3297898 | -0.71 | 1.84E-04 | 0.026 | PREDICTED: Homo sapiens similar to Ubiquinol-cytochrome c reductase hinge protein (LOC729769), mRNA. | |
| **LOC643336** | ILMN_3282321 | -0.75 | 1.93E-04 | 0.026 | PREDICTED: Homo sapiens similar to hCG1985303 (LOC643336), mRNA. | |
| **RNF150** | ILMN_1767446 | 0.81 | 2.22E-04 | 0.026 | Homo sapiens ring finger protein 150 (RNF150), mRNA. | |
| **PRDX3** | ILMN_2395974 | -0.72 | 2.44E-04 | 0.027 | Homo sapiens peroxiredoxin 3 (PRDX3), nuclear gene encoding mitochondrial protein, transcript variant 1, mRNA. | |
| **GPR37L1** | ILMN_1782944 | 0.83 | 2.63E-04 | 0.027 | Homo sapiens G protein-coupled receptor 37 like 1 (GPR37L1), mRNA. | |
| **VPS29** | ILMN_1704619 | -0.72 | 2.84E-04 | 0.027 | Homo sapiens vacuolar protein sorting 29 homolog (S. cerevisiae) (VPS29), transcript variant 1, mRNA. | |
| **LOC100129086** | ILMN_3236481 | -0.83 | 2.85E-04 | 0.027 | PREDICTED: Homo sapiens similar to HIG1 domain family, member 1A (LOC100129086), mRNA. | |
| **RPS21** | ILMN_1800573 | -0.74 | 3.11E-04 | 0.027 | Homo sapiens ribosomal protein S21 (RPS21), mRNA. | |
| **HSPA1A** | ILMN_1789074 | 1.18 | 3.11E-04 | 0.027 | Homo sapiens heat shock 70kDa protein 1A (HSPA1A), mRNA. | |
|  | ILMN_1878029 | 0.71 | 3.53E-04 | 0.028 | Homo sapiens cDNA FLJ34585 fis, clone KIDNE2008758 | |
| **BEX5** | ILMN_1806473 | -0.95 | 3.78E-04 | 0.028 | Homo sapiens brain expressed, X-linked 5 (BEX5), mRNA. | |
| **LOC100129685** | ILMN_3259223 | -0.72 | 4.13E-04 | 0.029 | PREDICTED: Homo sapiens hypothetical protein LOC100129685 (LOC100129685), mRNA. | |
| **COX7B** | ILMN_2184049 | -0.71 | 4.46E-04 | 0.029 | Homo sapiens cytochrome c oxidase subunit VIIb (COX7B), nuclear gene encoding mitochondrial protein, mRNA. | |
| **C10orf85** | ILMN_1665207 | 0.95 | 4.78E-04 | 0.030 | Homo sapiens chromosome 10 open reading frame 85 (C10orf85), mRNA. | |
| **MOCS2** | ILMN_2274923 | -0.71 | 5.98E-04 | 0.031 | Homo sapiens molybdenum cofactor synthesis 2 (MOCS2), transcript variant 1, mRNA. | Molybdenum cofactor deficiency (#252150) |
| **ARID5B** | ILMN_1721626 | 0.72 | 6.20E-04 | 0.032 | Homo sapiens AT rich interactive domain 5B (MRF1-like) (ARID5B), mRNA. | |
| **HSPB1** | ILMN_1674236 | 1.14 | 7.42E-04 | 0.033 | Homo sapiens heat shock 27kDa protein 1 (HSPB1), mRNA. | Charcot-Marie-Tooth disease, axonal type, 2F (#606595) |
| **LOC727865** | ILMN_3276209 | -0.73 | 7.66E-04 | 0.034 | PREDICTED: Homo sapiens misc_RNA (LOC727865), miscRNA. | |
| **SYN1** | ILMN_2407703 | 0.72 | 8.40E-04 | 0.034 | Homo sapiens synapsin I (SYN1), transcript variant Ia, mRNA. | Epilepsy, X-linked, with variable learning disabilities and behavior disorders (#300491) |
| **RERGL** | ILMN_3243185 | -0.73 | 1.43E-03 | 0.042 | Homo sapiens RERG/RAS-like (RERGL), mRNA. | |
| **DARC** | ILMN_1723684 | 0.80 | 1.79E-03 | 0.047 | Homo sapiens Duffy blood group, chemokine receptor (DARC), transcript variant 2, mRNA. | |
| **TUBB2B** | ILMN_1680874 | 0.71 | 2.00E-03 | 0.048 | Homo sapiens tubulin, beta 2B (TUBB2B), mRNA. | Asymmetric polymicrogyria (#610031) |

The top differentially expressed genes between autistic and control brain are listed at a FDR <5% and a log_2_-fold change of >0.7. P-values were adjusted by the method of Benjamini and Hochberg. OMIM mendelian disorders were listed if applicable. Gene description was from Illumina annotation. FC, fold change; OMIM, Online Mendelian Inheritance in Man.
