# Supplementary material for: Brain Transcriptional and Epigenetic Associations with Autism
Source: PLoS One. 2012 Sep 12;7(9):e44736. doi: 10.1371/journal.pone.0044736 (PMC3440365; doi:10.1371/journal.pone.0044736)
Supplement: Table S4 — Probes involved in oxidative phosphorylation differentially expressed between autistic and control brain after controlling for brain region. The top 300 differentially expressed probes between autistic and control brain at a FDR <5% included 17 oxidative phosphorylation genes identified in Ingenuity Pathway Analysis (pathway enrichment for oxidative phosphorylation p = 5.8E-14; Fisher’s exact test). P-values were adjusted by the method of Benjamini and Hochberg. OMIM mendelian disorders were listed if applicable. FC, fold change; OMIM, Online Mendelian Inheritance in Man. (DOC) [file pone.0044736.s008.doc]

**Table S4. Probes involved in oxidative phosphorylation differentially expressed between autistic and control brain after controlling for brain region.**

| **Gene symbol** | **Illumina probe ID** | **Log FC** | **Adjusted**  **p-value** | **Entrez Gene Name** | | **OMIM** |
| --- | --- | --- | --- | --- | --- | --- |
| **ATP5E** | ILMN_3261197 | -0.602 | 2.74E-02 | ATP synthase, H+ transporting, mitochondrial F1 complex, epsilon subunit | | Mitochondrial complex V deficiency (#614053) |
| **ATP5G1** | ILMN_1712430 | -0.515 | 2.20E-02 | ATP synthase, H+ transporting, mitochondrial Fo complex, subunit C1 (subunit 9) | | |
| **ATP5L** | ILMN_2079285 | -0.726 | 2.00E-02 | ATP synthase, H+ transporting, mitochondrial Fo complex, subunit G | | |
| **COX11** | ILMN_1666280 | -0.565 | 2.71E-02 | COX11 cytochrome c oxidase assembly homolog (yeast) | | |
| **COX7A2L** | ILMN_3237665 | -0.453 | 2.55E-02 | cytochrome c oxidase subunit VIIa polypeptide 2 like | | |
| **CYC1** | ILMN_1815115 | -0.385 | 2.35E-02 | cytochrome c-1 | | |
| **NDUFA2** | ILMN_3243890 | -0.553 | 2.20E-02 | NADH dehydrogenase (ubiquinone) 1 alpha subcomplex, 2, 8kDa | | Leigh syndrome (#256000) |
| **NDUFA6** | ILMN_3238269 | -0.408 | 2.58E-02 | NADH dehydrogenase (ubiquinone) 1 alpha subcomplex, 6, 14kDa | | |
| **NDUFA9** | ILMN_1760741 | -0.427 | 2.58E-02 | NADH dehydrogenase (ubiquinone) 1 alpha subcomplex, 9, 39kDa | Mitchondrial complex I deficiency (#256000) | |
| **NDUFA11** | ILMN_2175712 | -0.649 | 2.55E-02 | NADH dehydrogenase (ubiquinone) 1 alpha subcomplex, 11, 14.7kDa | | Mitochondrial complex I deficiency (#252010) |
| **NDUFB3** | ILMN_2119945 | -0.568 | 2.35E-02 | NADH dehydrogenase (ubiquinone) 1 beta subcomplex, 3, 12kDa | Mitochondrial complex I deficiency  (#603839) | |
| **NDUFB7** | ILMN_1813604 | -0.508 | 2.44E-02 | NADH dehydrogenase (ubiquinone) 1 beta subcomplex, 7, 18kDa | | |
| **NDUFS3** | ILMN_1756355 | -0.312 | 2.58E-02 | NADH dehydrogenase (ubiquinone) Fe-S protein 3, 30kDa (NADH-coenzyme Q reductase) | | Leigh syndrome (#256000) |
| **NDUFV2** | ILMN_2086417 | -0.330 | 2.71E-02 | NADH dehydrogenase (ubiquinone) flavoprotein 2, 24kDa | | Mitochondrial complex I deficiency (#252010) |
| **PPA2** | ILMN_1687785 | -0.688 | 2.73E-02 | pyrophosphatase (inorganic) 2 | | |
| **UQCR10** | ILMN_2366714 | -0.611 | 2.70E-02 | ubiquinol-cytochrome c reductase, complex III subunit X | | |
| **UQCRQ** | ILMN_1666471 | -0.531 | 2.75E-02 | ubiquinol-cytochrome c reductase, complex III subunit VII, 9.5kDa | | Mitochondrial complex III deficiency (#124000) |

The top 300 differentially expressed probes between autistic and control brain at a FDR <5% included 17 oxidative phosphorylation genes identified in Ingenuity Pathway Analysis (pathway enrichment for oxidative phosphorylation p = 5.8E-14; Fisher’s exact test). P-values were adjusted by the method of Benjamini and Hochberg. OMIM mendelian disorders were listed if applicable. FC, fold change; OMIM, Online Mendelian Inheritance in Man.
