# Supplementary material for: Brain Transcriptional and Epigenetic Associations with Autism
Source: PLoS One. 2012 Sep 12;7(9):e44736. doi: 10.1371/journal.pone.0044736 (PMC3440365; doi:10.1371/journal.pone.0044736)
Supplement: Table S5 — Probes associated with ribosomes differentially expressed between autistic and control brain after controlling for brain region. The top 300 differentially expressed probes between autistic and control brain at a FDR <5% included 12 ribosomal/protein translation genes identified in Ingenuity Pathway Analysis (pathway enrichment for EIF2 signaling p = 1.0E-6; Fisher’s exact test). P-values were adjusted by the method of Benjamini and Hochberg. OMIM mendelian disorders were listed if applicable. FC, fold change; OMIM, Online Mendelian Inheritance in Man. (DOC) [file pone.0044736.s009.doc]

**Table S5. Probes associated with ribosomes differentially expressed between autistic and control brain after controlling for brain region.**

| **Gene symbol** | **Illumina probe ID** | **Log_2_-FC** | **Adjusted p-value** | **Entrez Gene Name** | **OMIM** |
| --- | --- | --- | --- | --- | --- |
| **EIF2AK2** | ILMN_1706502 | 0.679 | 2.20E-02 | eukaryotic translation initiation factor 2-alpha kinase 2 | |
| **EIF2B3** | ILMN_3237907 | -0.389 | 2.20E-02 | eukaryotic translation initiation factor 2B, subunit 3 gamma, 58kDa | Leukoencephalopathy with vanishing white matter (#603896) |
| **EIF3B** | ILMN_1715636 | 0.271 | 2.55E-02 | eukaryotic translation initiation factor 3, subunit B | |
| **EIF3K** | ILMN_1694057 | -0.520 | 2.00E-02 | eukaryotic translation initiation factor 3, subunit K | |
| **FAU** | ILMN_1664614 | -0.390 | 2.55E-02 | Finkel-Biskis-Reilly murine sarcoma virus (FBR-MuSV) ubiquitously expressed | |
| **PPP1CA** | ILMN_1695827 | -0.436 | 2.55E-02 | protein phosphatase 1, catalytic subunit, alpha isozyme | |
| **RPL8** | ILMN_1811433 | -0.729 | 2.35E-02 | ribosomal protein L8 | |
| **RPL34** | ILMN_1706873 | -0.686 | 2.35E-02 | ribosomal protein L34 | |
| **RPL10** | ILMN_3280565 | -0.735 | 2.20E-02 | ribosomal protein L10 | Autism, susceptibility to, X-linked 5 (#300847) |
| **RPL10A** | ILMN_1808041 | -0.725 | 2.55E-02 | ribosomal protein L10a | |
| **RPS21** | ILMN_1800573 | -0.738 | 2.74E-02 | ribosomal protein S21 | |
| **RPS29** | ILMN_2415722 | -0.457 | 2.58E-02 | ribosomal protein S29 | |

The top 300 differentially expressed probes between autistic and control brain at a FDR <5% included 12 ribosomal/protein translation genes identified in Ingenuity Pathway Analysis (pathway enrichment for EIF2 signaling p = 1.0E-6; Fisher’s exact test). P-values were adjusted by the method of Benjamini and Hochberg. OMIM mendelian disorders were listed if applicable. FC, fold change; OMIM, Online Mendelian Inheritance in Man.
