# Supplementary material for: Brain Transcriptional and Epigenetic Associations with Autism
Source: PLoS One. 2012 Sep 12;7(9):e44736. doi: 10.1371/journal.pone.0044736 (PMC3440365; doi:10.1371/journal.pone.0044736)
Supplement: Table S6 — Differentially expressed genes in autism outlier cerebellar vs. age-matched controls. The top 300 DE probes at a FDR of <5% were consolidated into a non-redundant, mappable list of 75 genes. Log2-fold change in autism is reported for our dataset. There were six directly overlapping DE genes from Voineagu et al. and Garbett et al. top gene lists from temporal cortex, which was statistically significant (p = 1.4E-9, OR = 67 [95%CI: 23–170]; Fisher’s exact test). (DOC) [file pone.0044736.s010.doc]

**Table S6. Differentially expressed genes in autism outlier cerebellar vs. age-matched controls.**

| **Gene symbol** | **ILLUMINA_ID** | **Name** | **Ginsberg et al. log_2_-FC** | **Garbett et al.** | **Voineagu et al.** |
| --- | --- | --- | --- | --- | --- |
| **ACOT7** | ILMN_2332250 | acyl-CoA thioesterase 7 | 3.080106 |  |  |
| **AKR1C3** | ILMN_1713124 | aldo-keto reductase family 1, member C3 (3-alpha hydroxysteroid dehydrogenase, type II) | 5.620282 |  |  |
| **ALB** | ILMN_1782939 | Albumin | 4.67348 |  |  |
| **ALDH1A3** | ILMN_1807439 | aldehyde dehydrogenase 1 family, member A3 | 2.894003 |  |  |
| **ALPP** | ILMN_1693789 | alkaline phosphatase, placental (Regan isozyme) | -0.50208 |  |  |
| **ANXA2** | ILMN_2409167 | annexin A2 pseudogene 3; annexin A2; annexin A2 pseudogene 1 | 5.29992 |  |  |
| **ANXA2P1** | ILMN_2041101 | annexin A2 pseudogene 3; annexin A2; annexin A2 pseudogene 1 | 4.942727 |  |  |
| **BEX5** | ILMN_1806473 | brain expressed, X-linked 5 | 0.538141 |  |  |
| **BLZF1** | ILMN_2106658 | basic leucine zipper nuclear factor 1 | -0.32303 |  |  |
| **C5orf28** | ILMN_2150465 | chromosome 5 open reading frame 28 | 0.635824 |  |  |
| **C8orf37** | ILMN_2091084 | chromosome 8 open reading frame 37 | -1.07419 |  |  |
| **C8orf45** | ILMN_2113738 | chromosome 8 open reading frame 45 | 0.035146 |  |  |
| **CALB1** | ILMN_1760199 | calbindin 1, 28kDa | 0.332514 |  |  |
| **CBLN1** | ILMN_1712461 | Cerebellin 1 precursor | 4.747151 |  |  |
| **CDAN1** | ILMN_2401906 | congenital dyserythropoietic anemia, type I | -0.86521 |  |  |
| **CEBPD** | ILMN_1782050 | CCAAT/enhancer binding protein (C/EBP), delta | 2.362793 |  |  |
| **CIRBP** | ILMN_1674661 | cold inducible RNA binding protein | 1.03139 |  |  |
| **COL4A1** | ILMN_1653028 | collagen, type IV, alpha 1 | 1.633958 | up | up |
| **CRCP** | ILMN_2381537 | CGRP receptor component | -0.31328 |  |  |
| **CRYM** | ILMN_1690179 | crystallin, mu | 2.132389 |  |  |
| **CSDA** | ILMN_1782788 | cold shock domain protein A; cold shock domain protein A pseudogene 1 | 0.80722 | up | up |
| **CYP26B1** | ILMN_1812297 | cytochrome P450, family 26, subfamily B, polypeptide 1 | 5.520677 |  |  |
| **DDIT4** | ILMN_1661599 | DNA-damage-inducible transcript 4 | 1.900814 |  |  |
| **DEM1** | ILMN_2117569 | defects in morphology 1 homolog (S. cerevisiae) | -0.04504 |  |  |
| **FKBP14** | ILMN_2150294 | FK506 binding protein 14, 22 kDa | -0.59657 |  |  |
| **GAD1** | ILMN_2292646 | Glutamate decarboxylase 1 (brain, 67kDa) | 0.065591 |  |  |
| **GFAP** | ILMN_1697176 | glial fibrillary acidic protein | 4.285565 |  |  |
| **GJB6** | ILMN_2226223 | gap junction protein, beta 6, 30kDa | 3.538415 |  |  |
| **HSPC268** | ILMN_2078547 | chromosome 7 open reading frame 55 | 0.730069 |  |  |
| **ICAM2** | ILMN_1786823 | intercellular adhesion molecule 2 | 5.610456 |  |  |
| **IFITM2** | ILMN_1673352 | Interferon induced transmembrane protein 2 (1-8D) | 3.923136 | up | up |
| **IFITM3** | ILMN_1805750 | Interferon induced transmembrane protein 3 (1-8U) | 4.544813 | up | up |
| **KIAA1751** | ILMN_2415979 | KIAA1751 | 0.162014 |  |  |
| **LILRB1** | ILMN_2316974 | leukocyte immunoglobulin-like receptor, subfamily B (with TM and ITIM domains), member 1 | -1.27795 |  |  |
| **LOC399942** | ILMN_1765701 | hypothetical gene supported by AF081484; NM_006082; tubulin, alpha 1b | 1.208017 |  |  |
| **LOC642817** | ILMN_1695034 | hypothetical LOC642817 | 2.534995 |  |  |
| **LOC645385** | ILMN_1720745 | similar to Heterogeneous nuclear ribonucleoprotein A1 (Helix-destabilizing protein) (Single-strand binding protein) (hnRNP core protein A1) (HDP-1) (Topoisomerase-inhibitor suppressed) | 2.30226 |  |  |
| **LOC648210** | ILMN_1732074 | heterogeneous nuclear ribonucleoprotein A1-like 3; similar to heterogeneous nuclear ribonucleoprotein A1; heterogeneous nuclear ribonucleoprotein A1 pseudogene 2; heterogeneous nuclear ribonucleoprotein A1; heterogeneous nuclear ribonucleoprotein A1 pseudogene | 2.919533 |  |  |
| **LRRC32** | ILMN_2129161 | leucine rich repeat containing 32 | 2.072526 |  |  |
| **MCM8** | ILMN_1798581 | minichromosome maintenance complex component 8 | -1.28628 |  |  |
| **MT1E** | ILMN_2173611 | metallothionein 1L (gene/pseudogene); metallothionein 1E; metallothionein 1 pseudogene 3; metallothionein 1J (pseudogene) | 5.525155 |  |  |
| **MT1M** | ILMN_1657435 | metallothionein 1M | 4.629913 |  |  |
| **MT2A** | ILMN_1686664 | metallothionein 2A | 4.533607 |  |  |
| **MYBPC1** | ILMN_1752075 | myosin binding protein C, slow type | 5.05599 |  |  |
| **NDRG3** | ILMN_2385097 | NDRG family member 3 | 3.91509 |  |  |
| **NLRP8** | ILMN_2075794 | NLR family, pyrin domain containing 8 | -0.47728 |  |  |
| **NSF** | ILMN_1680687 | N-ethylmaleimide-sensitive factor | 4.369811 |  |  |
| **NTRK2** | ILMN_1714067 | neurotrophic tyrosine kinase, receptor, type 2 | 2.877401 |  |  |
| **OCIAD1** | ILMN_2330495 | OCIA domain containing 1 | -0.72098 |  |  |
| **PDE4C** | ILMN_1761277 | phosphodiesterase 4C, cAMP-specific (phosphodiesterase E1 dunce homolog, Drosophila) | -0.67758 |  |  |
| **PENK** | ILMN_1726711 | proenkephalin | 3.237514 |  |  |
| **PLIN5** | ILMN_2209993 | lipid storage droplet protein 5 | -0.85774 |  |  |
| **POFUT1** | ILMN_2276758 | protein O-fucosyltransferase 1 | -0.88986 |  |  |
| **PPA2** | ILMN_2342455 | pyrophosphatase (inorganic) 2 | 0.279746 |  |  |
| **PTGR2** | ILMN_2105253 | prostaglandin reductase 2 | 0.822961 |  |  |
| **RASL12** | ILMN_1806403 | RAS-like, family 12 | 4.469629 |  |  |
| **RASSF6** | ILMN_2352245 | Ras association (RalGDS/AF-6) domain family member 6 | 2.764078 |  |  |
| **RN7SK** | ILMN_1739423 | RNA, 7SK small nuclear | 2.951866 |  |  |
| **S100A10** | ILMN_2046730 | S100 calcium binding protein A10 | 4.617054 | up | up |
| **SEMA3E** | ILMN_2154322 | sema domain, immunoglobulin domain (Ig), short basic domain, secreted, (semaphorin) 3E | -0.97026 |  |  |
| **SHROOM4** | ILMN_2206188 | shroom family member 4 | 0.415919 |  |  |
| **SLC25A18** | ILMN_1754864 | solute carrier family 25 (mitochondrial carrier), member 18 | 4.013694 |  |  |
| **SPTLC1** | ILMN_2249473 | serine palmitoyltransferase, long chain base subunit 1 | -1.04681 |  |  |
| **TAGLN** | ILMN_1778668 | Transgelin | 3.672374 |  |  |
| **TAGLN2** | ILMN_2090105 | Transgelin 2 | 4.844188 | up | up |
| **TGM2** | ILMN_1705750 | transglutaminase 2 (C polypeptide, protein-glutamine-gamma-glutamyltransferase) | 3.015408 |  |  |
| **TM4SF1** | ILMN_1770338 | transmembrane 4 L six family member 1 | 5.300362 |  |  |
| **TXNIP** | ILMN_1697448 | thioredoxin interacting protein | 1.038257 |  |  |
| **VPS24** | ILMN_1683827 | vacuolar protein sorting 24 homolog (S. cerevisiae); ring finger protein 103 | 3.635218 |  |  |
| **XRCC2** | ILMN_2204909 | X-ray repair complementing defective repair in Chinese hamster cells 2 | -0.41067 |  |  |
| **YRDC** | ILMN_1736008 | yrdC domain containing (E. coli) | 0.884947 |  |  |
| **ZNF483** | ILMN_2279834 | zinc finger protein 483 | 0.549688 |  |  |
| **ZNF486** | ILMN_1805668 | zinc finger protein 486 | -0.89783 |  |  |
| **ZNF652** | ILMN_2155322 | zinc finger protein 652 | -0.17175 |  |  |
| **ZNF69** | ILMN_2176882 | zinc finger protein 69 | -0.88767 |  |  |

The top 300 DE probes at a FDR of <5% were consolidated into a non-redundant, mappable list of 75 genes. Log_2_-fold change in autism is reported for our dataset. There were six directly overlapping DE genes from Voineagu et al. and Garbett et al. top gene lists from temporal cortex, which was statistically significant (p=1.4E-9, OR=67 [95%CI: 23-170]; Fisher’s exact test).
