# Supplementary material for: Brain Transcriptional and Epigenetic Associations with Autism
Source: PLoS One. 2012 Sep 12;7(9):e44736. doi: 10.1371/journal.pone.0044736 (PMC3440365; doi:10.1371/journal.pone.0044736)
Supplement: Table S7 — Previously implicated genes for DNA methylation abnormalities were not differentially methylated between autistic and control BA19 or cerebellar cortex by microarray analysis. Probes for BCL2, MECP2, OXTR, RORA, and UBE3A were compared between 9 matched autistic and control BA19 or cerebellar cortical samples. P-values are for tests analogous to two-tailed, paired t-tests. Differences are for group mean M-values (M-values are logit transformed beta-values, where beta-value is approximately the proportion of methylation at a particular locus). No probes were statistically significant, controlling for multiple comparisons (adjusted p-value = 0.002). All nucleotide positions are for the NCBI36/hg18 genome build. (DOC) [file pone.0044736.s011.doc]

**Table S7. Previously implicated genes for DNA methylation abnormalities were not differentially methylated between autistic and control BA19 or cerebellar cortex by microarray analysis.**

| **Gene symbol** | **Probe ID** | **Chromosome** | **Nucleotide position** | **Difference (BA19)** | **P-value (BA19)** | **Difference (cerebellar)** | **P-value (cerebellar)** |
| --- | --- | --- | --- | --- | --- | --- | --- |
| **BCL2** | cg01803238 | 18 | 59137006 | -0.12 | 0.434 | 0.170188 | 0.325644 |
| **BCL2** | cg03813215 | 18 | 59137659 | -0.02 | 0.784 | -0.13386 | 0.125659 |
| **BCL2** | cg05927017 | 18 | 59139135 | 0.09 | 0.621 | -0.04953 | 0.681317 |
| **BCL2** | cg08223235 | 18 | 59054814 | 0.08 | 0.633 | -0.09148 | 0.422197 |
| **BCL2** | cg08554462 | 18 | 59137891 | -0.10 | 0.200 | -0.14151 | 0.152912 |
| **BCL2** | cg09752703 | 18 | 59137602 | 0.00 | 0.941 | -0.00056 | 0.994764 |
| **BCL2** | cg11255230 | 18 | 59138788 | 0.22 | 0.009 | -0.10686 | 0.295265 |
| **BCL2** | cg11330108 | 18 | 59137095 | -0.19 | 0.207 | -0.13336 | 0.383585 |
| **BCL2** | cg12459502 | 18 | 59055217 | -0.01 | 0.964 | -0.17658 | 0.270315 |
| **BCL2** | cg14455307 | 18 | 59138409 | 0.07 | 0.574 | -0.03128 | 0.847891 |
| **BCL2** | cg17602451 | 18 | 59136625 | 0.10 | 0.516 | -0.03943 | 0.642248 |
| **BCL2** | cg21602520 | 18 | 59136360 | 0.15 | 0.398 | -0.04813 | 0.713271 |
| **BCL2** | cg23756272 | 18 | 59055398 | 0.12 | 0.206 | 0.017332 | 0.909497 |
| **BCL2** | cg24408313 | 18 | 59139445 | -0.01 | 0.967 | -0.11506 | 0.50067 |
| **BCL2** | cg25059899 | 18 | 59055308 | 0.14 | 0.166 | -0.13564 | 0.432815 |
| **MECP2** | cg00981643 | X | 153016902 | -0.19 | 0.211 | 0.247594 | 0.133679 |
| **MECP2** | cg11479591 | X | 153015370 | -0.03 | 0.769 | -0.05599 | 0.668487 |
| **OXTR** | cg23391006 | 3 | 8786279 | -0.33 | 0.003 | 0.042499 | 0.713268 |
| **OXTR** | cg25140571 | 3 | 8786437 | 0.08 | 0.291 | -0.1326 | 0.358414 |
| **RORA** | cg13301933 | 15 | 59308731 | -0.07 | 0.296 | -0.00902 | 0.939481 |
| **RORA** | cg27167601 | 15 | 59309215 | 0.24 | 0.160 | 0.138806 | 0.451869 |
| **UBE3A** | cg00792740 | 15 | 23235581 | -0.03 | 0.723 | -0.11236 | 0.436798 |
| **UBE3A** | cg02517489 | 15 | 23234168 | -0.04 | 0.721 | 0.324901 | 0.055861 |
| **UBE3A** | cg05881762 | 15 | 23235942 | -0.14 | 0.554 | -0.45892 | 0.03532 |
| **UBE3A** | cg12177023 | 15 | 23234344 | 0.00 | 0.976 | -0.03942 | 0.727791 |
| **UBE3A** | cg12572011 | 15 | 23235178 | 0.01 | 0.962 | 0.1786 | 0.275138 |
| **UBE3A** | cg18766912 | 15 | 23235002 | 0.09 | 0.702 | -0.06464 | 0.689043 |
| **UBE3A** | cg23681243 | 15 | 23235181 | -0.18 | 0.159 | -0.22859 | 0.20334 |
| **UBE3A** | cg27458888 | 15 | 23235671 | 0.15 | 0.285 | -0.0704 | 0.713637 |

Probes for BCL2, MECP2, OXTR, RORA, and UBE3A were compared between 9 matched autistic and control BA19 or cerebellar cortical samples. P-values are for tests analogous to two-tailed, paired t-tests. Differences are for group mean M-values (M-values are logit transformed beta-values, where beta-value is approximately the proportion of methylation at a particular locus). No probes were statistically significant, controlling for multiple comparisons (adjusted p-value=0.002). All nucleotide positions are for the NCBI36/hg18 genome build.
