# Supplementary material for: Brain Transcriptional and Epigenetic Associations with Autism
Source: PLoS One. 2012 Sep 12;7(9):e44736. doi: 10.1371/journal.pone.0044736 (PMC3440365; doi:10.1371/journal.pone.0044736)
Supplement: Table S8 — Primers used for real-time reverse-transcriptase quantitative PCR. Primers were designed to span introns or lie on an exon-exon junction. See Methods for description of assay conditions. (DOCX) [file pone.0044736.s012.docx]

**Table S8. Primers used for real-time reverse-transcriptase quantitative PCR.**

| **Gene symbol** | **Forward primer** | **Reverse primer** | **Accession number** | **Amplicon length (bp)** |
| --- | --- | --- | --- | --- |
| **A2BP1** | CCTGCATCCTTATGGCGTGCCT | TACCGTTCTGCGGGGGAGCA | [NM_145891.2](http://www.ncbi.nlm.nih.gov/entrez/viewer.fcgi?db=nucleotide&id=215272406) | 146 |
| **BCL2** | GTGGAGAGCGTCAACCGGGAGA | GGGCCGTACAGTTCCACAAAGGC | [NM_000633.2](http://www.ncbi.nlm.nih.gov/entrez/viewer.fcgi?db=nucleotide&id=72198188) | 137 |
| **CEBPD** | TGCGGAGCCTGCGCCCTTCTA | GCTGAAGTCGATGGCGCTCTCG | [NM_005195.3](http://www.ncbi.nlm.nih.gov/entrez/viewer.fcgi?db=nucleotide&id=125661056) | 133 |
| **CNTNAP1** | CCGCTGCGTGGAGCGCTATA | GCGCAGCGCTGACTGTAGGT | [NM_003632.2](http://www.ncbi.nlm.nih.gov/entrez/viewer.fcgi?db=nucleotide&id=221316606) | 139 |
| **CREB1** | ATCCGGGCCGTGAACGAAAGC | CTGTGGCTGGGCTTGAACTGTCA | [NM_004379.3](http://www.ncbi.nlm.nih.gov/entrez/viewer.fcgi?db=nucleotide&id=215490111) | 150 |
| **GABRB1** | CCCGTCGACGTTGGGATGCG | GGGTGAGGTTCAGTGGGATTCCAGA | [NM_000812.3](http://www.ncbi.nlm.nih.gov/entrez/viewer.fcgi?db=nucleotide&id=194097326) | 145 |
| **GAPDH** | GCCGCATCTTCTTTTGCGTCGC | TGGTGACCAGGCGCCCAATAC | [NM_002046.3](http://www.ncbi.nlm.nih.gov/entrez/viewer.fcgi?db=nucleotide&id=83641890) | 120 |
| **IFITM2** | ATGTCGTCTGGTCCCTGTTC | CCAACCATCTTCCTGTCCC | [NM_006435.2](http://www.ncbi.nlm.nih.gov/entrez/viewer.fcgi?db=nucleotide&id=151101190) | 103 |
| **MOBP** | GCGCCTGCCAGAAGACCAGATTG | GCAATGAGTCCAGGAGCCGAGG | [NM_182935.2](http://www.ncbi.nlm.nih.gov/entrez/viewer.fcgi?db=nucleotide&id=109150417) | 110 |
| **RORA** | GAGGCAAGAGTGCCGTGGTCAA | CAGCCTGCTCAGGGAGCTACAG | [NM_002943.3](http://www.ncbi.nlm.nih.gov/entrez/viewer.fcgi?db=nucleotide&id=213385297) | 129 |
| **RTN4** | GGAGCTGCAAAGCAGATCGTGAC | GGCTGGCACCAAACACCACTCC | [NM_007008.2](http://www.ncbi.nlm.nih.gov/entrez/viewer.fcgi?db=nucleotide&id=47519538) | 122 |
| **UBE3A** | GGAGTGGCTTGCAGGATGGAGAA | TGCAGCTGCTCGCTTCATTCGG | [NM_000462.3](http://www.ncbi.nlm.nih.gov/entrez/viewer.fcgi?db=nucleotide&id=332000023) | 102 |

Primers were designed to span introns or lie on an exon-exon junction. See Methods for description of assay conditions.
