# Supplementary material for: Brain Transcriptional and Epigenetic Associations with Autism
Source: PLoS One. 2012 Sep 12;7(9):e44736. doi: 10.1371/journal.pone.0044736 (PMC3440365; doi:10.1371/journal.pone.0044736)
Supplement: Table S9 — Primers used for pyrosequencing of bisulfite-converted DNA. (DOC) [file pone.0044736.s013.doc]

**Table S9. Primers used for pyrosequencing of bisulfite-converted DNA.**

| **Gene symbol** | **Sense primer (5’ to 3’)** | **Anti-sense primer (5’ to 3’)** | **Sequencing primer (5’ to 3’)** | **Amplicon length (bp)** | **Annealing temperature (°C)** |
| --- | --- | --- | --- | --- | --- |
| **OXTR** | GTTTYGAAAGTTTTGGAATTTTTGAT | AAACCCRAATAAAAATAATAAAATACCT | AAAGTTTTGGAATTTTTGATT | 158 | 55 |
| **UBE3A** | GGYGAGGGTTTTAGGTYGGG | CAAACCACCTCCTCTTCCCAATAACC | GAGGGTTTTAGGTYGGGA | 155 | 65 |
| **CEBPD** | AGGTTGTTATTTYGTTGGGTTTAGTTT | AAATCCCCAAAACCRAAAAAAAAACCC | TTTTTTAGTTTYGGTTG | 233 | 60 |
| **MECP2** | TTATAAGTTTTGTTATTTTTAGGGGA | TACCTCTAAATTCCATATATTCTTTC | TTTTGTTATTTTTAGGGGAT | 181 | 56 |
| **RORA** | ATAACCAAACCCRCCTCTTATCCCCT | AAAAAAAAAAAAGAAGGAGAAG | CACACACACRTCCACCCAA | 153 | 58 |
| **BCL2** | TGTTAGYGAAGGTGTYGGGGT | CACCCTTTCTCCTCCTCCTAATC | GGGTTTYGGGTTTTTTTTGT | 136 | 60 |
